# Structure of 3-mercaptopropionic acid dioxygenase with a substrate analog reveals bidentate substrate binding at the iron center

**DOI:** 10.1016/j.jbc.2021.100492

**Published:** 2021-03-01

**Authors:** Nicholas J. York, Molly M. Lockart, Sinjinee Sardar, Nimesh Khadka, Wuxian Shi, Ronald E. Stenkamp, Jianye Zhang, Philip D. Kiser, Brad S. Pierce

**Affiliations:** 1 Department of Chemistry & Biochemistry, University of Alabama, Tuscaloosa, Alabama, USA; 2 Department of Chemistry & Biochemistry, The University of Texas at Arlington, Arlington, Texas, USA; 3 Department of Pharmacology, Case Western Reserve University, Cleveland, Ohio, USA; 4 National Synchrotron Light Source-II, Brookhaven National Laboratory, Upton, New York, USA; 5 Departments of Biological Structure and Biochemistry, University of Washington, Seattle, Washington, USA; 6 Department of Ophthalmology, School of Medicine, University of California, Irvine, Irvine, California, USA; 7 Department of Physiology & Biophysics, School of Medicine, University of California, Irvine, Irvine, California, USA; 8 Research Service, VA Long Beach Healthcare System, Long Beach, California, USA

**Keywords:** thiol dioxygenase, nonheme iron, oxygenase, competitive inhibition, structure, DFT, computational modeling, iron-nitrosyl, FT-EPR, HYSCORE, 2MA, 2-mercaptoanaline, 3HPA, 3-hydroxypropionic acid, 3MDO, 3-mercaptopropionic acid dioxygenase, 3MPA, 3-mercaptopropionic acid, 3SPA, 3-sulfinopropionic acid, ADO, cysteamine dioxygenase, *Av*3MDO, Azotobacter vinelandii 3MDO, CA, cysteamine (2-aminoethanethiol), CDO, cysteine dioxygenase, CSA, cysteine sulfinic acid, CW, continuous wave, CYS, L-cysteine, DFT, density-functional theory, DNIC, dinitrosyl iron complex, EPR, electron paramagnetic resonance, ET, ethanethiol, FT, Fourier transform, H-bond, hydrogen bond, HEPES, 4-(2-hydroxyethyl)-1-piperazineethanesulfonic acid, HT, hypotaurine, HYSCORE, hyperfine sublevel correlation spectroscopy, IPTG, isopropyl β-D-1-thiogalactopyranoside, MSDO, mercaptosuccinate dioxygenase, Pa3MDO, *Pseudomonas aeruginosa* 3MDO, PCO, plant cysteine oxidases, RnCDO, *Rattus norvegicus* CDO, SHY, Ser-His-Tyr, TPTZ, 2,4,6-tripyridyl-s-triazine, XRD, X-ray diffraction

## Abstract

Thiol dioxygenases are a subset of nonheme iron oxygenases that catalyze the formation of sulfinic acids from sulfhydryl-containing substrates and dioxygen. Among this class, cysteine dioxygenases (CDOs) and 3-mercaptopropionic acid dioxygenases (3MDOs) are the best characterized, and the mode of substrate binding for CDOs is well understood. However, the manner in which 3-mercaptopropionic acid (3MPA) coordinates to the nonheme iron site in 3MDO remains a matter of debate. A model for bidentate 3MPA coordination at the 3MDO Fe-site has been proposed on the basis of computational docking, whereas steady-state kinetics and EPR spectroscopic measurements suggest a thiolate-only coordination of the substrate. To address this gap in knowledge, we determined the structure of *Azobacter vinelandii* 3MDO (*Av*3MDO) in complex with the substrate analog and competitive inhibitor, 3-hydroxypropionic acid (3HPA). The structure together with DFT computational modeling demonstrates that 3HPA and 3MPA associate with iron as chelate complexes with the substrate-carboxylate group forming an additional interaction with Arg168 and the thiol bound at the same position as in CDO. A chloride ligand was bound to iron in the coordination site assigned as the O_2_-binding site. Supporting HYSCORE spectroscopic experiments were performed on the (3MPA/NO)-bound *Av*3MDO iron nitrosyl (*S* = 3/2) site. In combination with spectroscopic simulations and optimized DFT models, this work provides an experimentally verified model of the *Av*3MDO enzyme–substrate complex, effectively resolving a debate in the literature regarding the preferred substrate-binding denticity. These results elegantly explain the observed 3MDO substrate specificity, but leave unanswered questions regarding the mechanism of substrate-gated reactivity with dioxygen.

Thiol dioxygenases are a subset of nonheme mononuclear iron oxygenases that catalyze the O_2_-dependent oxidation of thiol-bearing substrates to yield the corresponding sulfinic acid. Among this group, cysteine dioxygenase (CDO) (1, 2, 3, 4, 5) and cysteamine dioxygenase (ADO) (6, 7) are responsible for the biosynthesis of cysteine sulfinic acid (CSA) and hypotaurine (HT), respectively, which are precursors for the biosynthesis of taurine (1). Among bacteria, a number of other thiol dioxygenases have been identified, including mercaptosuccinate dioxygenase (MSDO) (8) and 3-mercaptopropionate dioxygenase (3MDO) (9, 10, 11, 12, 13, 14). Plant cysteine oxidases (PCO) catalyze the formation of an *N*-terminal cysteine sulfinic acid within ERF-VII transcription factors to initiate N-end rule degradation (15, 16). A similar function has recently been proposed for mammalian ADO in controlling regulators of G protein signaling (17).

Over the past decade, this class of enzymes has attracted considerable attention as imbalances in L-cysteine (CYS) metabolism are associated with neurological diseases (5, 18, 19). This observation suggests a correlation between impaired sulfur metabolism, oxidative stress, and neurodegenerative disease (20, 21). Consequently, enzymes involved in sulfur oxidation and transfer are increasingly being evaluated as potential drug targets (22, 23, 24, 25).

Across the domains of life, structurally characterized thiol dioxygenases share two major features: (1) a mononuclear nonheme iron active site coordinated by three protein-derived histidine residues and (2) a conserved sequence of outer Fe-coordination sphere amino acids (Ser-His-Tyr), the latter being adjacent to the iron site (∼3 Å). By analogy to the chymotrypsin-like serine proteases, the Ser-His-Tyr (“SHY”) network was previously referred to as a “*catalytic triad*” (5, 13, 14, 26, 27, 28, 29, 30). However, these residues appear to enhance the catalytic rate and efficiency but are not required for activity (31, 32, 33, 34). Consequently, we simply refer to this hydrogen-bonding network as the “SHY” motif. In eukaryotic CDOs, the “SHY” motif tyrosine (Tyr157) is covalently cross-linked with an adjacent cysteine residue (C93) to yield a C93-Y157 pair (35, 36), whereas bacterial forms utilize an unmodified Tyr-residue at this position.

As shown in Figure 1*A*, the substrate-bound mammalian CDO reveals a bidentate coordination of CYS to the mononuclear Fe-site *via* thiolate and neutral amine (32, 37, 38). Both kinetic and spectroscopic studies demonstrate additional interactions between the CYS-bound Fe-site and outer sphere residues (Tyr157 and Arg60) within the CDO active site (27, 31). These multiple points of interaction are likely responsible for the high substrate specificity exhibited by this enzyme (35). To illustrate, cysteamine [2-aminoethanethiol, (CA)] is structurally similar to CYS, lacking only the α-carboxylate group. It is therefore reasonable that this substrate analogue could coordinate to the CDO Fe-site in a similar bidentate fashion as CYS. However, relative to CYS, a 10,000-fold decrease in *k*_*cat*_/*K*_*M*_ was reported in steady-state reactions with CA (35). Therefore, removal of the CYS carboxylate has a profound impact on the formation of the CDO ES complex. More dramatically, no activity whatsoever is observed in reactions with similarly sized thiol substrates lacking an amine functional group. This observation demonstrates that coordination of the substrate amine to the CDO Fe-site is required for activity. By contrast, 3MDO (Fig. 1*B*) is capable of accommodating a variety of thiol-bearing substrates, such as 3-mercaptopropionic acid (3MPA), CYS, and CA to catalyze dioxygenation over a broad pH range (10, 11, 13, 14). While a modest decrease in *k*_*cat*_ is observed in reactions with CA [0.29 ± 0.08 s^−1^] relative to 3MPA and CYS [1.0 ± 0.1 s^−1^], the vast difference in catalytic efficiency (*k*_*cat*_/*K*_*M*_) identifies 3MPA as the preferred substrate for MDO (14). Since 3MPA lacks an amine functional group, the first coordination sphere for the *Av*3MDO Fe-site cannot be equivalent to CDO.

**Figure 1:**
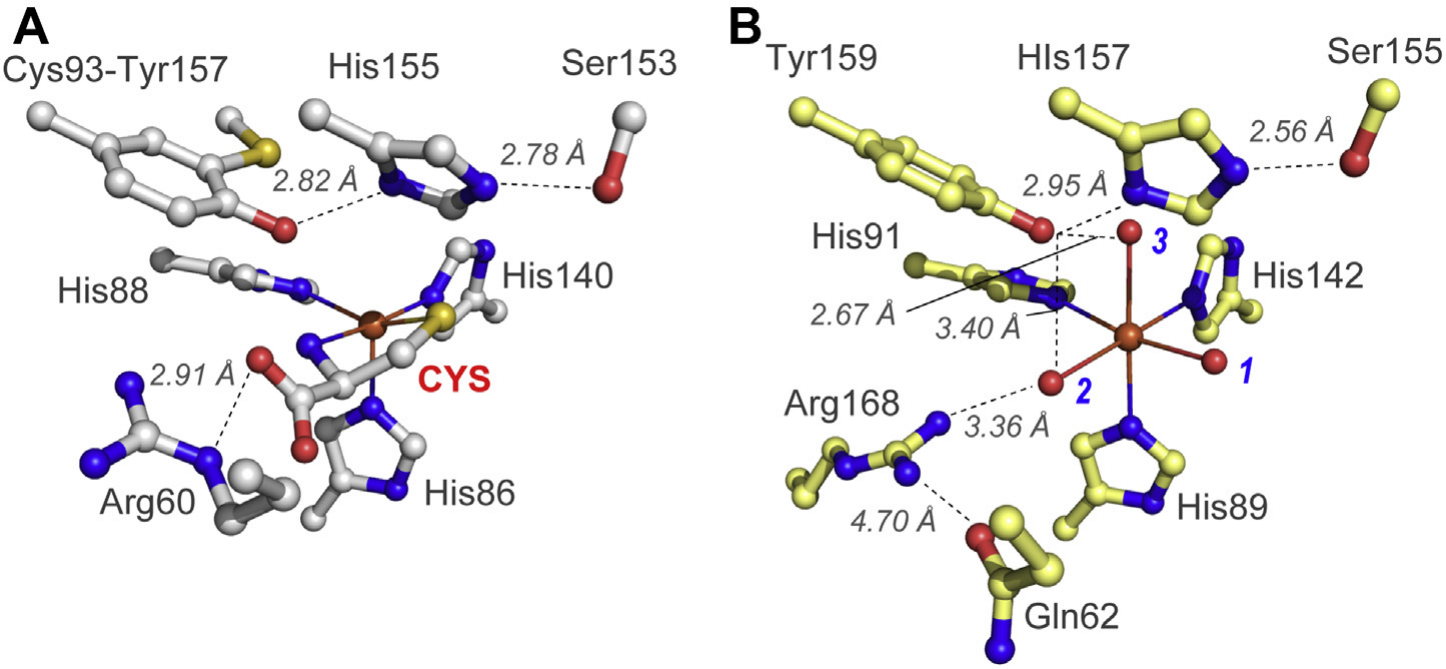
**Structural comparison of mammalian CDO and *Pseudomonas aeruginosa* 3MDO active site.***A*, 1.6 Å X-ray diffraction structure of *CYS*-bound *Rattus norvegicus* CDO (PDB accession code 4IEV) (90). Selected atomic distances are designated by *dashed lines*. *B*, 2.14 Å X-ray diffraction structure of the *Pseudomonas aeruginosa* 3MDO active site (PDB 4TLF) (11). Fe-coordinated solvent ligands designated (*1*–*3*) for clarity.

To date, structures for the 3MPA-bound enzyme have been unavailable; however, two models for substrate binding at the Fe-site have been proposed. A bidentate model for 3MPA coordination to the Fe-site *via* substrate thiol and carboxylate functional groups was recently proposed by Jameson and Karplus (9). In this report, docking of 3MPA into the *Pseudomonas aeruginosa* 3MDO (*Pa*3MDO) crystal structure led to the conclusion that a salt bridge formed between the Arg168 guanidinium group and 3MPA carboxylate was catalytically essential (9, 10). Structurally, this model closely matches the CYS-bound mammalian CDO, although the relevant Arg group is in a position nonhomologous to that of mammalian CDO. The proposed salt bridge provided a reasonable explanation for the decreased affinity of the enzyme for amino-bearing substrates. However, the instability of *Pa*3MDO Arg168 variants precluded experimental validation of this model by site-directed mutagenesis.

Alternatively, we have previously argued that 3MPA Fe-coordination occurs *via* thiolate only based on the results of two key experiments (12). First, pH-dependent kinetic data for *Av*3MDO-catalyzed reactions with a substrate lacking a carboxyl group (cysteamine, CA) reveal only a modest decrease in *k*_*cat*_-values [*v*_*0*_/[E], 0.29 ± 0.08 s^−1^] relative to reactions performed with 3MPA and CYS [1.0–1.2 s^−1^] (14). Since both CDO and 3MDO exhibit an obligate-ordered binding of organic substrate prior to molecular oxygen, we conclude that Fe-coordination of the substrate–carboxylate group is not required to produce the O_2_-activating enzyme–substrate (ES) complex. Second, experiments using the O_2_-mimetic nitric oxide corroborate this argument. EPR spectroscopy verifies formation of a substrate-bound iron-nitrosyl species using thiol substrates lacking carboxylate groups [such as CA and ethanethiol (ET)] (14). Therefore, direct Fe-coordination of a substrate carboxylate is not required for converting the Fe(II) site into an NO-reactive state. These results clearly demonstrate that direct carboxylate coordination to the iron center is not obligatory to trigger oxygen activation.

In order to address this discrepancy in 3MDO substrate-binding models, we present the structure of *Av3*MDO in complex with the substrate analog and competitive inhibitor, 3-hydroxypropionic acid (3HPA). Using the *Av*3MDO-3HPA complex as a starting point, density functional theory (DFT) computations were performed to model the coordination of the native 3MPA substrate. Supporting HYSCORE experiments performed on the (3MPA/NO)-bound *Av*3MDO confirm the NO-binding site and corroborate bidentate 3MPA coordination in the substrate-bound iron-nitrosyl site. As suggested by Jameson and Karplus, these experiments support bidentate coordination of 3MPA with the substrate carboxylate group forming an additional interaction with Arg168 and the thiol bound at the same position as CDO.

## Results

### Crystal structure of *Av*3MDO in complex with 3HPA

Following extensive sparse matrix screening, we identified a condition giving rise to well-diffracting, rod-shaped *Av*3MDO crystals. The optimized crystals diffracted X-rays to ∼2.2 Å resolution and belonged to space group *P*3_1_ (Table S1). Structure determination was initially complicated by the presence of tetartohedral twinning and pseudosymmetry (see Experimental procedures section for further details), but eventually the structure was solved by molecular replacement and refined against reflections extending to 2.25 Å resolution (Table S1). The polypeptide is clearly resolved except for the extreme *N*- and *C* termini, which are omitted from the final model. The asymmetric unit of the *Av*3MDO crystals consists of 12 monomers arranged in pairs of C_2_ symmetric dimers. The moderately hydrophobic dimer interface buries an average of ∼1260 Å^2^ (1199–1322 Å^2^ depending on the specific dimer pair) or ∼13% of the total monomer surface area (39) and is structurally similar to the dimers observed for 3MDOs from other bacteria (11, 26), all suggesting that it represents a physiologically relevant assembly. This finding contrasts with cysteine dioxygenases, which are monomeric proteins (37, 40).

**Figure 2:**
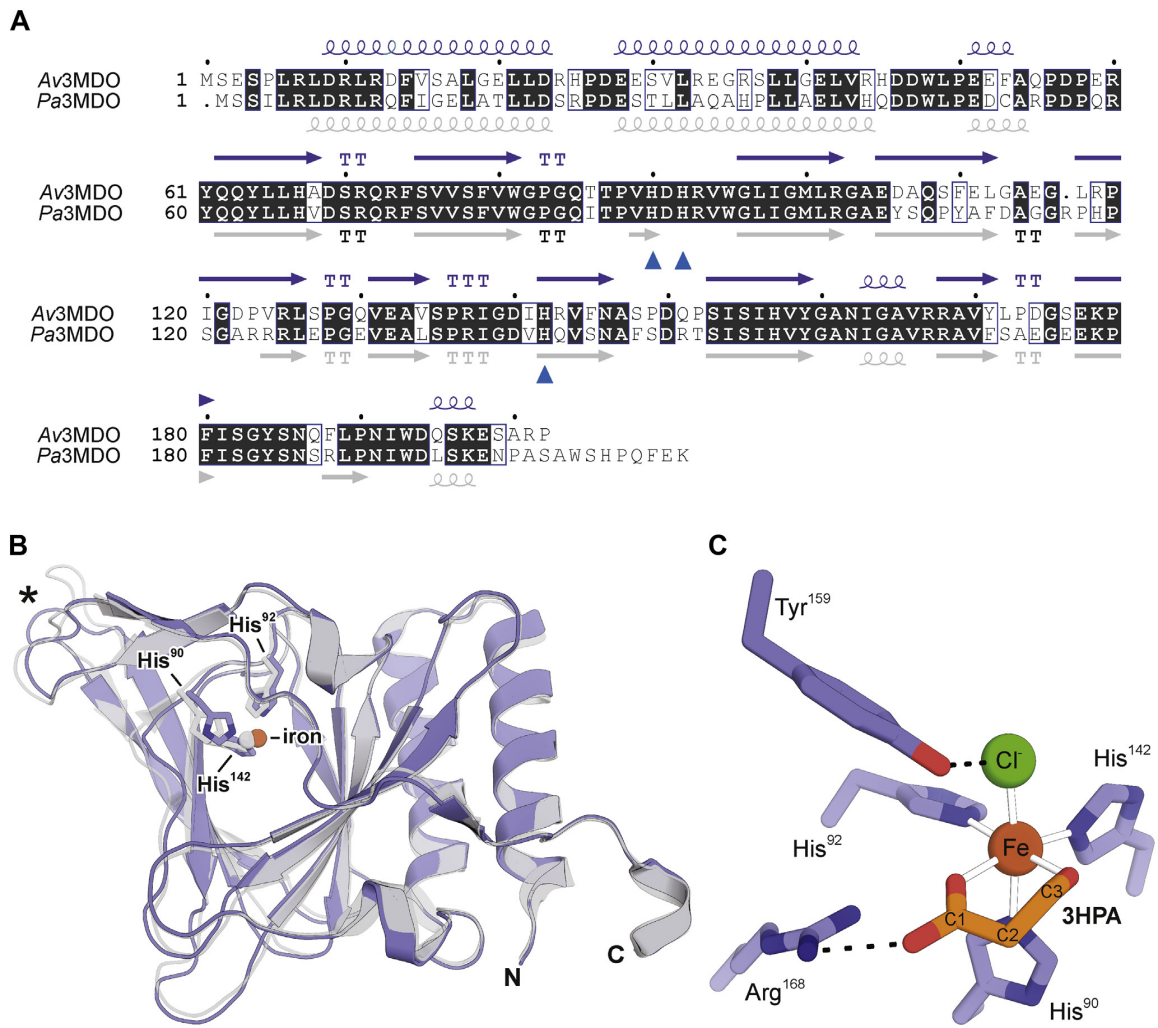
**Structure of *Av*3MDO and its comparison to Pa3MDO.***A*, sequence alignment of *Av*3MDO and *Pa*3MDO with secondary structural elements colored in *slate* and *gray*, respectively. *Curly lines*, *arrows*, and “T” represent α-helices, β-strands, and β-turns, respectively. The iron-binding His residues are marked by *blue arrows*. *B*, structural superposition of *Av*3MDO (*slate*) and *Pa*3MDO (*gray*). Note the slight shift in His90, His92, and the iron cofactor as well as the conformational difference at the loop marked by an *asterisk*, which is due to a one residue deletion between residue 116 and 117 in *Av*3MDO. N and C mark the N- and C-termini, respectively. The superposition was performed using the “super” command in PyMOL. *C*, structure of the *Av*3MDO iron center (*brown sphere*) in complex with 3HPA (*orange sticks*) and chloride (*green sphere*). *Dashed lines* indicate H-bonding interactions.

*Av*3MDO exhibits the classic thiol dioxygenase cupin fold consisting of three *N*-terminal alpha-helices followed by a series of 11 β-strands forming the cupin β-barrel structure that houses the enzymatic active site (Fig. 2*A*). Owing to the near uniformity in structure between the 12 monomers (Cα RMSD <0.4 Å), chain B was used as the representative monomer for structural comparisons. Among 3MDOs of known structure, *Av*3MDO exhibits greatest sequence and structural similarity to the 3MDO enzyme from *P. aeruginosa* (*Pa*3MDO, PDB accession code: 4TLF) with 70% sequence identity and a Cα RMSD of ∼1 Å. The largest main chain difference is found within the Leu113-Leu117 β hairpin where the insertion of an Arg in *Pa*3MDO results in a 5.8 Å shift at the tips of the β hairpins (Fig. 2*B*, *asterisk*). Like in *Pa*3MDO and other thiol dioxygenases (11, 26, 28), the iron center of *Av*3MDO is coordinated by three His residues (90, 92, and 142) contained within the conserved cupin motifs (Fig. 2*C*). Although the *Av*3MDO sample used for crystallization was ∼36% loaded with iron, the iron *B*-factors are similar to those of the surrounding His ligands when the metal is modeled at full occupancy, indicating the metal-loaded enzyme selectively crystallized (Table S1). Additionally, the metal is modeled as an Fe(III) ion given that a bulk of the iron in the enzyme sample used for crystallization was in the ferric oxidation state. However, some portion of the iron may have been photoreduced to Fe(II) during data collection. Analysis of residual electron density maps revealed two strong features near the *Av*3MDO iron center, which could not be successfully modeled with aquo ligands as was the case for *Pa*3MDO (11) (Fig. S1). As described in detail in the Experimental procedures section, we modeled these density features with chloride (*trans* to His90) and 3-hydroxypropionic acid (3HPA, *trans* to His 92 and 142), the latter being present as contaminant in the polyacrylate solution used for crystallization (Fig. 2*C*, Figs. S1 and S2).

3HPA binds to the iron center in a bidentate fashion *via* its hydroxyl and carboxylate oxygen atoms with metal–ligand bond distances of ∼2.1 to 2.2 Å (depending on the particular monomer). The carboxylate group simultaneously engages in an ionic interaction with Arg168, which is situated adjacent to the iron center. The 3HPA carboxylate oxygen is located ∼3.1 Å from the hydroxyl group of Tyr159, which is part of the “SHY” motif and is known to influence substrate binding to the iron center (12). Notably, we do not observe a significant interaction of 3HPA with Gln63, the distinguishing residue of the “Gln-type” thiol dioxygenases (26). These results indicate that the carboxylate-interacting Arg residue is relocated from position 61, as found in Arg-type thiol dioxygenases, to a nonhomologous position in the “Gln-type” thiol dioxygenases, as was previously suggested based on docking studies of 3MPA in *Pa*3MDO (9).

**Figure 3:**
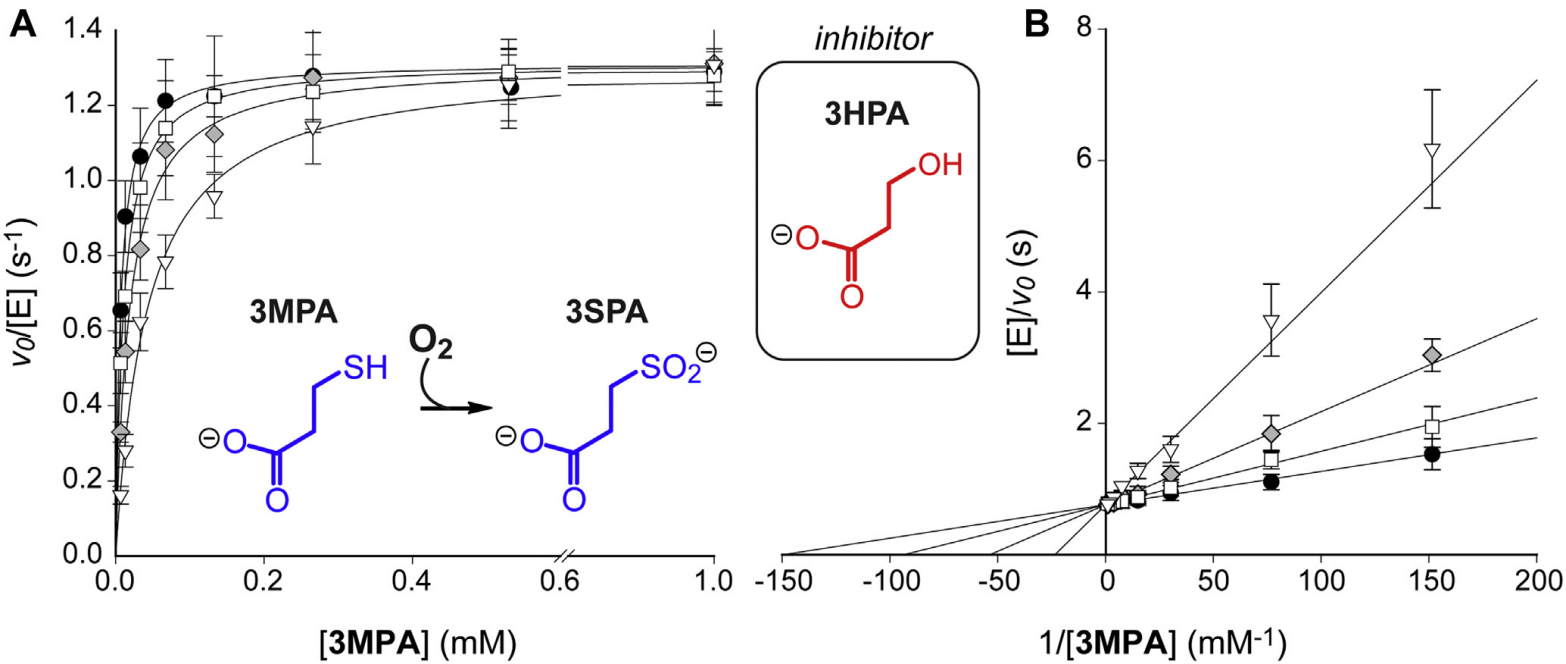
**3-hydroxypropionic acid (3HPA) inhibition of *Av*3MDO-catalyzed 3MPA-reactions.** Kinetic data were collected in the presence of 167 μM (*white square*), 500 μM (*gray diamond*), and 1500 μM (*white triangle*) 3HPA for comparison to the uninhibited enzyme (*black circle*). SigmaPlot was used to globally fit enzyme kinetics in either Michaelis–Menten (*A*) or Lineweaver–Burk (*B*) fashion assuming a fully competitive model of inhibition. The resulting least-square fits (*solid-lines*) are overlaid on kinetic data to obtain values for *k*_*cat*_, *K*_*M*_, and *K*_*I*_, as well as the error associated with each parameter [1.31 ± 0.01 s^−1^, 6.7 ± 0.4 μM, and 280 ± 26 μM, respectively]. Michaelis–Menten results (*A*) are presented with a gap ranging from 0.6 to 0.8 mM 3MPA to avoid data crowding at low concentration.

Despite the close structural resemblance of 3HPA to the native 3MPA substrate of 3MDOs (Fig. 3, *inset*), the influence of this compound on 3MDO activity has, to our knowledge, never been examined. Given its observed mode of binding to the iron center, we hypothesized that 3HPA could act as an inhibitor of *Av3MDO*. We tested this by carrying out *Av*3MDO steady-state assays in the presence of 3HPA (Fig. 3). We previously demonstrated that the initial rate of *Av*3MDO-catalyzed 3MPA reactions is independent of oxygen concentration down to ∼25 μM (13). Therefore, atmospheric oxygen concentration is sufficient to saturate the enzyme kinetics. Moreover, the consumption of oxygen per 3-sulfinopropionic acid (3SPA) produced is essentially stoichiometric (14). Crucially, no oxygen is consumed upon mixing enzyme with excess of 3HPA (10 mM). Therefore, the initial rate of oxygen consumption was used to monitor enzymatic inhibition as a function of inhibitor concentration. A Lineweaver–Burk plot of the activity data revealed an inhibition constant (K_i_) of 280 ± 26 μM and intersection point at the ordinate consistent with the behavior of a classic competitive inhibitor. These results establish that 3HPA is an effective substrate analog and that its mode of binding to *Av*3MDO likely mirrors that of 3MPA.

Chloride binds to the iron center *trans* to His90 at a distance of ∼2.4 Å and simultaneously contacts the hydroxyl moiety of Tyr159 forming a 2.7 Å ion–dipole interaction (Fig. 2*C*). This corresponds to the binding site for solvent 3 in the resting *Pa*3MDO structure (Fig. 1*B*). To distinguish this site from the other solvent-bound positions, we refer to this as the *axial* Fe-coordination site henceforth. Similar binding of chloride has been observed in structures of CDO (27, 41) but not in other “Gln-type” thiol dioxygenases determined to date. Activity studies carried out in the presence of increasing Cl^−^ show a modest degree of inhibition (30%) at the 100 mM concentration found in the crystal (Fig. S3). However, EPR experiments demonstrated that Cl^−^ concentration has no impact on the extent of NO binding to the 3MPA-bound enzyme; therefore, it is unlikely that this chloride inhibition reflects competition for O_2_ binding at the iron center (*data not shown*). It is possible that Cl^−^ coordination is only significant for the ferric form of the enzyme, owing to its ability to provide charge balance to the Fe(III)-site. Regardless, the presence of an anionic ligand at the putative O_2_-binding site of the enzyme supports the idea that Tyr159 could facilitate the formation of an Fe(III)-superoxo intermediate during the *Av*3MDO catalytic cycle *via* hydrogen bonding (12).

### *Av*3MDO active site accessibility

**Figure 4:**
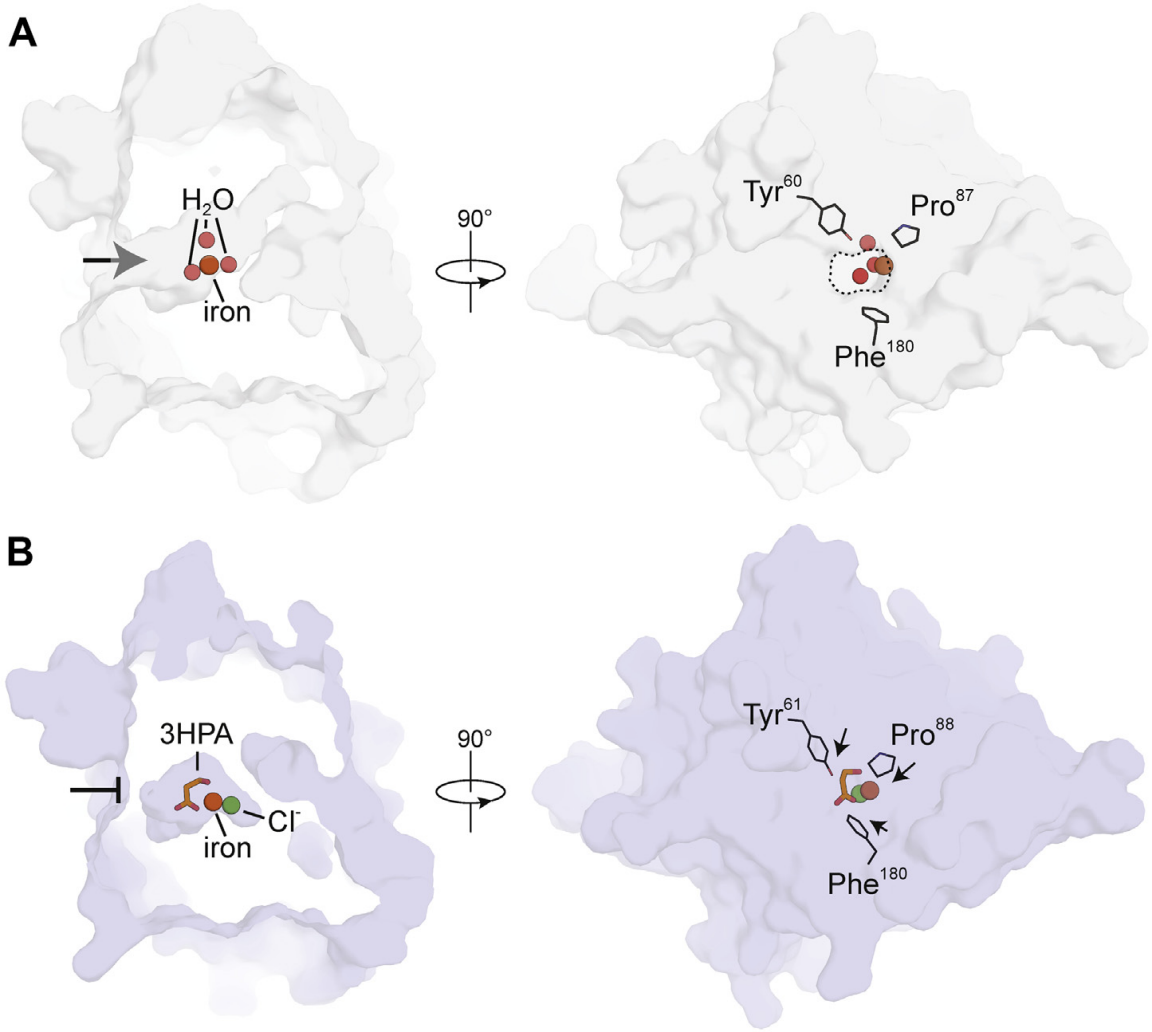
**An occluded active site channel in 3HPA-bound *Av*3MDO.** In contrast to the resting-state structure of *Pa*3MDO (*A*) in which the active site is readily accessible to organic substrate through a passageway marked by an *arrow on the left view* and a *dashed circle on the right view*, the active site of *Av*3MDO (*B*) is completely sealed to bulk solvent due to positional differences in Tyr61, Pro88, and Phe180 (directionality differences with respect to the corresponding residues in *Pa*3MDO indicated by *arrows*). The structures are shown as Connolly surfaces.

**Figure 5:**
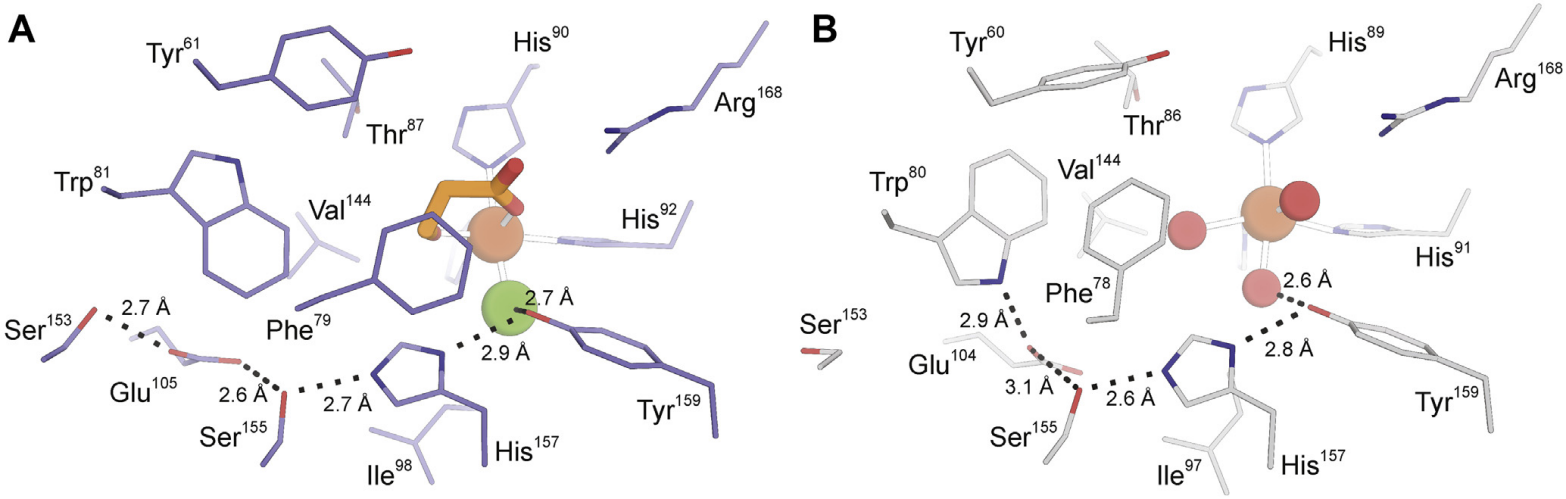
**Active site differences observed between 3HPA-bound *Av*3MDO and Pa3MDO.** Comparison of the 3HPA-bound structure of *Av*3MDO (*A*) and the resting-state structure of *Pa*3MDO (*B*) reveals active site conformational differences in Phe79/Phe78 and Trp81/Trp80 and a rearrangement of the “SHY” H-bonding network with Glu105 interacting with Ser153 and Ser155 in the *Av*3MDO structure.

MDO homologs of *Av*3MDO, including *Pa*3MDO, exhibit active site openings on one side of the cupin beta barrel that presumably serves as the passageway for organic substrate diffusion (Fig. 4*A*, *left*). However, inspection of the *Av*3MDO structure revealed that its active site is completely sealed off to the bulk solvent (Fig. 4*B*, *left*). In comparison to the homologous residues of *Pa*3MDO, residues Tyr61, Pro88, and Phe180 of *Av*3MDO are shifted inward toward each other to occlude the potential passageway (Fig. 4, *A* and *B*, *right panels*). We observed additional variation within the active site of *Av*3MDO as compared with *Pa*3MDO including rotamer differences for Phe79 and Trp81 that were found in all 12 copies within the asymmetric unit (Fig. 5). Notably, the difference in Trp81 rotameric state results in different arrangements of the “SHY” motif between *Av*3MDO (Fig. 5*A*) and *Pa*3MDO (Fig. 5*B*). In the *Pa*3MDO structure, the Ser155 residue of this motif forms a hydrogen bond with Glu104, which simultaneously interacts with the Trp80 indole NH group. The alternative conformation of Trp81 as observed in the *Av3*MDO structure results in a ∼2.2 Å shift in Glu105 toward Ser153, which also has an altered conformation in the *Av*3MDO structure (Fig. 5), resulting in a new H-bond interaction with Ser153. These collective changes produce an extension of the “SHY” motif in *Av*3MDO.

Because intrinsic structural differences between *Av*3MDO and *Pa*3MDO are expected to be small owing to their high sequence similarity (Fig. 2*A*), we considered the possibility that the observed active site differences could arise from the bound substrate mimetic in *Av*3MDO. To test this hypothesis, we identified alternative crystallization conditions to allow investigation of the resting-state structure of *Av*3MDO. Following further sparse matrix screening, we identified a crystallization condition containing a nonpolyacrylate precipitant (pentaerythritol propoxylate) as well as sodium thiocyanate that produced yellowish, rod-shaped crystals. The crystals diffracted X-rays to ∼3 Å resolution and belonged to space group *P*6_1_22 with a pair of dimers (same conserved interface described above) in the asymmetric unit. Strong difference density in the equatorial positions of the iron center was adequately explained by two water molecules while the axial position contained an elongated difference peak that was too strong to represent only water or chloride (Fig. S4*A*). Placement of thiocyanate, a well-known Fe(III)-binding ligand present at > 0.3 M concentration in the crystal mother liquor, at this position adequately accounted for the density with reasonable refined *B*-factors (Table S1). Because thiocyanate is an ambivalent ligand, test refinements were carried out with the anion bound *via* either its nitrogen or sulfur atoms. Analysis of difference maps and refined *B*-factors provided strong evidence that coordination is through the nitrogen atom, which is consistent with the coordination preferences of Fe(III), a hard Lewis acid, and small-molecule crystallography results for high-spin, nonheme iron model compounds (42). Although this structure is not a true “resting-state” form of *Av*3MDO, it allowed us to address the question of whether the active site closure results from 3HPA binding.

Contrary to our hypothesis, we observed that the active site has maintained its closed state in this new crystal form (Fig. S4*B*). Hence, we conclude that the closed conformation is energetically favorable *in crystallo* and that active site closure is not strictly coupled to 3HPA coordination. Notable differences between the two *Av*3MDO crystal forms included a ∼40° rotation of the Phe79 Cβ-Cγ bond away from the iron center in the 3HPA-bound structure as well as rotamer differences for Gln63 and Ile98, which all are likely a consequence of steric factors involving the different metal-bound ligands (Fig. S4*A*). By contrast, the conformation of Trp81 is identical in every copy within the asymmetric units of the two *Av*3MDO structures, suggesting it represents an intrinsic structural difference as compared with *Pa*3MDO.

### Computational modeling of the *Av*3MDO-3MPA complex

The structure of the *Av*3MDO-3HPA complex revealed a bidentate coordination of 3HPA to the Fe-site through the hydroxyl group and proximal oxygen of the carboxylate (Figs. 2*C* and 5*A*). Given the near structural equivalence of 3MPA and the competitive inhibitor 3HPA, a logical argument for bidentate 3MPA coordination within the enzymatic site can be made. Based on this hypothesis, we determined the optimal 3MDO Fe-site geometry for the bidentate *Av*3MDO-3MPA complex through DFT calculations using the coordinates from the 3HPA-bound *Av*3MDO crystal structure as a starting point. A truncated model for the inhibitor-bound active site, comprised of iron, His residues 90, 92, and 142, chloride, and Arg168, was generated and geometry-optimized with both 3HPA and 3MPA bound to the iron. Selected first-coordination sphere distances are presented in Table S2 for the *Av*3MDO-HPA complex, CYS-bound CDO, synthetic nonheme mononuclear iron model complexes, and our computational models. Although the bulk oxidation state observed for *Av*3MDO was ferric prior to crystallization, the possibility of reduction by the synchrotron source could not be ruled out.

The pKa of the 3HPA hydroxyl (14.4–15.1) is expected to be much higher than the 3MPA-thiol (∼8) (43). While it is unlikely that the Fe-bound 3HPA-hydroxyl is deprotonated, pKa values for metal-coordinated water can decrease by several pH units (44, 45). To corroborate the ionization state of the 3HPA inhibitor, optimized DFT models for the deprotonated (3HPA^2−^)- and protonated (3HPA^1−^)-hydroxyl group were made with ferric and ferrous iron for comparison to the crystallographic results (Table 1). While the overall structures were similar (Fig. S5), the protonated (3HPA^1−^)-bound model exhibited the lowest RMSD values relative to crystallographic coordinates. Moreover, optimized bond distances and angles closely match what is observed for the *Av*3MDO-3HPA complex. From this we conclude that the 3HPA-inhibitor coordinates bidentate to the Fe-site through carboxylate and neutral hydroxyl groups. For simplicity, all calculations and 3HPA-bound structures discussed henceforth refer solely to the 3HPA^1−^ form in which the hydroxyl group is protonated. However, the calculated structural perturbations incurred by altering the oxidation state of the iron (+II *versus* +III) site fall within the inherent error (∼0.1 Å) of the crystal structure. Therefore, neither oxidation state can be ruled out on the basis of these calculations.

**Table 1:** Selected distances (*top*) and angles (*bottom*) of the *Av*3MDO-3HPA complex (PDB accession code 6XB9) as compared with optimized DFT models of the iron active bound to 3HPA and 3MPA

Selected geometric parameters	6XB9	Fe(III) 3HPA^2−^	Fe(III) 3HPA^1−^	Fe(III) 3MPA	Fe(II) 3HPA^2−^	Fe(II) 3HPA^1−^	Fe(II) 3MPA
RMSD^a^	-	0.382	0.248	0.417	0.517	0.357	0.547
Distance (Å)							
Fe-O/S distance (Å)	2.16	1.87	2.23	2.32	1.91	2.26	2.33
Fe-O_(carb)_^b^ (Å)	2.17	2.14	1.97	2.08	2.55	2.25	2.51
Fe-Cl (Å)	2.36	2.36	2.28	2.36	2.60	2.38	2.47
Fe-His_(Ave)_	2.16	2.18	2.13	2.20	2.17	2.15	2.19
Fe-H90	2.15	2.17	2.17	2.19	2.18	2.16	2.20
Fe-H92	2.16	2.20	2.08	2.21	2.18	2.11	2.22
Fe-H142	2.16	2.17	2.13	2.19	2.16	2.19	2.15
Angles ∠ (°)							
Fe-O/S-C_α_	92.5	120.6	113.0	96.7	117.7	111.6	98.4
H90-Fe-Cl	175.7	171.8	174.0	169.3	169.6	171.5	166.3
H92-Fe-O/S	171.1	177.9	174.1	171.9	168.2	179.3	175.9
O_(carb)_-Fe-O/S	101.1	92.1	85.1	96.4	82.6	88.6	88.8
3HPA/3MPA C_1_-C_2_-C_3_	114.6	116.9	115.6	119.3	116.5	117.8	117.6

The 3HPA-inhibitor was modeled as both the protonated and deprotonated alcohol (3HPA^1−^ and 3HPA^2−^) for comparison.

a Hydrogens and all constrained atoms were excluded from RMSD calculations.

b Fe-O_(carb)_ designates the distance separating the Fe(III)-site from the 3HPA (or 3MPA) carboxylate O-atom.

**Figure 6:**
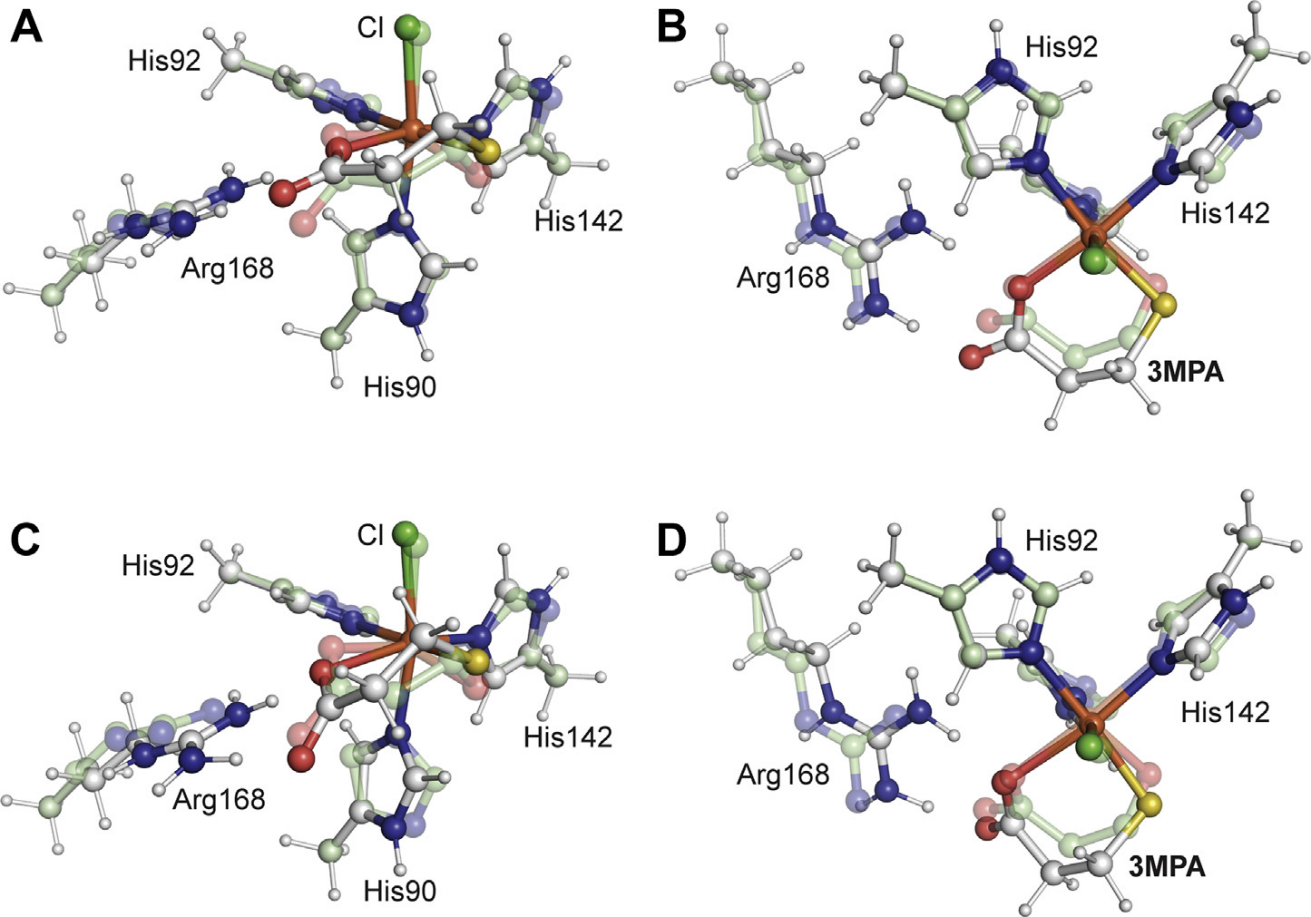
**Overlay of 3HPA-bound *Av*3MDO complex (*transparent*-*green*) with a DFT optimized model (*white*) of the 3MPA-bound *Av*3MDO Fe(III)-site (RMSD, 0.417 Å).** Shown from a side-on view (*A*) and following 90° rotation (*B*) through the bond of His142 (which is excluded from the image). These views are replicated in panels *C* and *D* for the ferrous optimized model. Selected distances, angles, and RMSD values are presented in Table 1 for comparison.

Given the ambiguous oxidation state of the iron site, 3MPA-bound structures were optimized in both the ferric and ferrous states. Figure 6 illustrates the Fe-sites for the *Av*3MDO-3HPA XRD structure and the DFT-optimized 3MPA-bound model. Selected bond distances and angles for both optimized structures are provided in Table 1 for comparison. DFT-optimized structures show a shorter bond length between the iron and 3HPA-hydroxyl than with the corresponding 3MPA-thiolate [2.23 *versus* 2.32 Å, respectively for ferric and 2.26 *versus* 2.33 Å for ferrous]. Similarly, the distance separating the substrate carboxylate O-atom from the Fe-site (Fe-O_carbox_) is closer for the ferric 3HPA-bound model [2.08 for 3MPA *versus* 1.97 Å for 3HPA]. The trend is more drastic in the ferrous state with 2.25 Å for 3HPA and 2.51 Å for 3MPA. The longer bond length for the 3MPA-bound structure can likely be attributed to (1) electrostatic interaction of the anionic carboxylate group with the adjacent cationic Arg168 and (2) inclusion of thee negative charges [Cl^−^ and 3HPA^2−^ (or 3MPA^2−^)] in the Fe(II) coordination sphere. However, charge neutrality is easily remedied by loss of a chloride ligand in the ferrous enzyme. For both ferric computational models, these distances are shorter than observed in the *Av3*MDO-3HPA complex (∼2.17 Å). By contrast, both ferrous models are longer, but well within the estimated coordinate error of the crystal structure (∼0.1 Å). Similarly, the average Fe-N_His_ and Fe-Cl bond lengths for the 3MPA-bound complex are largely invariant and any deviations observed fall well within the coordinate error of the *Av*3MDO-3HPA complex. For comparison, selected bond distances for thiolate-bound nonheme ferric thiol dioxygenase enzymes and model complexes are provided in Table S2.

Regardless of iron oxidation state (+II or +III), all optimized structures modeled (3HPA^2−^, 3HPA^1−^, and 3MPA) predict a bidentate Fe coordination *via* a single carboxylate O-atom and terminal oxide/hydroxyl or thiolate. As predicted by Aloi *et al.* (9), this places the substrate carboxylate in a favorable position for electrostatic stabilization by cationic Arg168. Taken together with the XRD structure of the *Av*3MDO-3HPA complex, these optimized DFT structures infer that the native 3MPA substrate coordinates to the *Av*3MDO iron site in a bidentate fashion as well.

### Modeling of the (3MPA/NO)-bound *Av*3MDO

**Figure 7:**
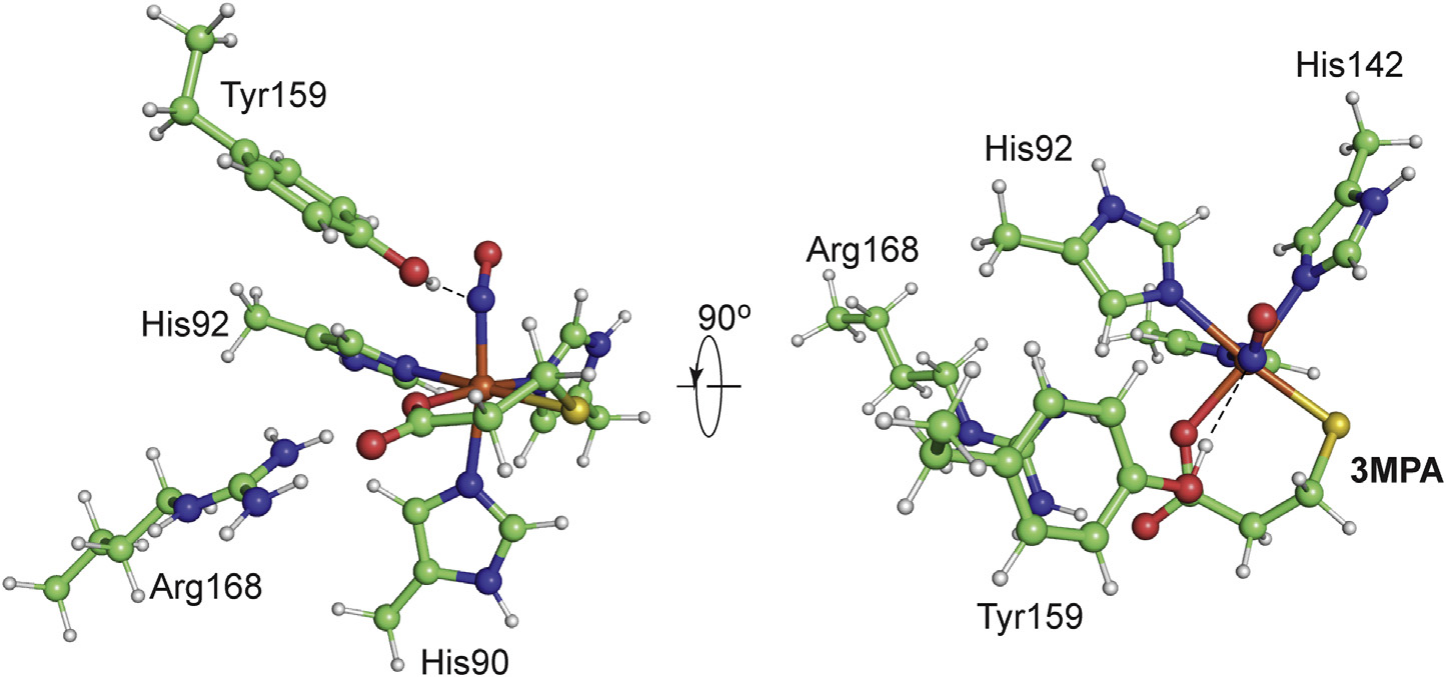
**The optimized structure of the (3MPA/NO)-bound *Av*3MDO active site.** The *left panel* displays a side view highlighting the axial nitric oxide ligand. The *right panel* rotates the structure to more clearly show bidentate coordination of 3MPA and Tyr159 hydrogen bond donation to the nitrogen of the NO ligand.

Nitric oxide is frequently used as a surrogate for molecular oxygen when characterizing nonheme iron oxidase/oxygenases. These experiments provide an excellent handle for EPR spectroscopy as the resulting iron-nitrosyl {FeNO}^7^ species is paramagnetic (*S* = 3/2). According to the Feltham–Enemark notation (46), the ground state *S* = 3/2 spin-manifold is produced by an antiferromagnetic coupling between a high-spin Fe(III) (*S* = 5/2) and a bound NO^−^ anion (*S* = 1). Moreover, given the similarity of the NO electronic structure to O_2_, the resulting (3MPA/NO)-bound *Av*3MDO ternary complex likely provides insight into transient iron-oxo species produced during native turnover with oxygen. The 3MPA-bound iron nitrosyl form of *Av*3MDO has been extensively characterized by EPR and Mössbauer spectroscopies (12, 14). Such data provides an opportunity to validate computational models by comparing predicted spectroscopic properties to experimental values. To this end, we studied whether bidentate iron coordination of 3MPA is retained in the (3MPA/NO)-bound ternary complex. Starting from the optimized *Av*3MDO-3MPA model, a new structure for the (3MPA/NO)-bound *Av*3MDO active site was modeled and optimized. As shown in Figure 7, nitric oxide was placed in putative oxygen-binding site *trans* to His90, essentially displacing the axial chloride in previous models. This positioning of NO is also consistent with previous EPR experiments, which suggest that hydrogen bonding from Tyr159 stabilizes NO binding within the active site (12). The outer sphere Tyr159 was included in optimized models to account for this interaction.

The precise orientation of hydrogen bonding between Tyr159 and Fe-bound nitric oxide is not known. Therefore, an energy surface (47, 48, 49) for Tyr159 hydrogen bonding was generated from the *Av*3MDO-3HPA complex to evaluate likely H-bond acceptors (Supporting Information). As shown in Fig. S6*C*, an energy minimum is observed for H-bond donation from Tyr159 to the Fe-bound Cl atom for both ferrous and ferric oxidation states. Potentially, substitution of the axial chloride ligand for NO could alter the direction of the Tyr159 H-bond to favor donation to H157. However, this would be inconsistent with the previous EPR and Mössbauer results. Moreover, as both chlorine and nitrosyl ligands have an equivalent formal charge (−1), a reasonable argument can be made that H-bond donation from Tyr159 would similarly favor axially bound NO over His157. Table S3 illustrates the influence of Tyr159 H-bond orientation on selected geometric parameters in the optimized (3MPA/NO)-bound *Av*3MDO Fe-site.

As an initial validation of the model, Mössbauer spectroscopic parameters were calculated for the optimized (3MPA/NO)-bound active site structures allowing comparison to the experimental values. As shown in Table S4, the isomer shift (δ) and quadrupole splitting (ΔE_Q_) observed for the (3MPA/NO)-bound enzyme are reasonably reproduced by all optimized models. In particular, ΔE_Q_ is highly sensitive to the nature and symmetry of ligands directly coordinated to the Fe-site. Thus, the closeness of calculated ΔE_Q_-values strongly supports the hypothesis of a bidentate 3MPA coordination within the (3MPA/NO)-bound enzyme. Overall, the ΔE_Q_ and asymmetry parameter (η) are best fit in optimized structures with Tyr159 donating a hydrogen bond to the N-atom of nitric oxide. However, the observed deviations among optimized structures largely fall within experimental error. Therefore, while the calculated Mössbauer parameters are entirely consistent with the optimized model shown in Figure 7, the orientation of Tyr159 hydrogen bond donation cannot be definitively assigned based solely on these results.

### HYSCORE of the (3MPA/NO)-bound *Av*3MDO

**Figure 8:**
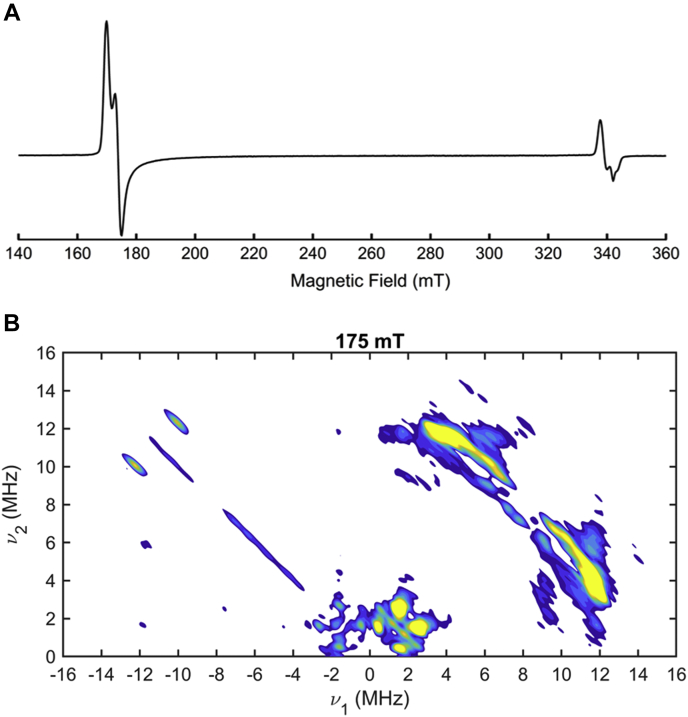
**CW EPR (*A*) and HYSCORE (*B*) spectra of (3MPA/NO)-bound *Av*3MDO iron-nitrosyl.** The instrumental parameters for (*A*) (previously reported) are described in detail elsewhere (12). The data in (*B*) were collected with the following instrumental parameters: microwave frequency, 9.78 GHz; field position of 175 mT; temperature, 5 K; pulse repetition rate, 1.25 kHz; τ, 120 ns. Additional instrumental parameters are described in detail in Experimental procedures.

As noted above, we have previously reported the characterization of iron-nitrosyl produced by treating 3MPA-bound *Av*3MDO with nitric oxide using continuous-wave (CW) EPR spectroscopy (12). The CW EPR spectrum, shown in Figure 8 (panel *A*), has observed *g*-values of 4.06, 3.96, and 2.01 consistent with a nearly axial (*E*/*D* = 0.008) *S* = 3/2 iron-nitrosyl site. Further, the measured axial zero field splitting (*D* = 10 ± 2 cm^−1^) confirms that this signal is derived from a transition within the ground |m_s_ = ± 1/2> doublet (38). These parameters are consistent with other spectroscopically and crystallographically characterized *S* = 3/2 iron-nitrosyl complexes (50, 51). Crucially, the observed CW spectrum is nearly homogeneous and exhibits only minor contributions from known dinitrosyl (DNIC) complexes localized near *g* = 2. As a result, this complex is ideal for further characterization by hyperfine sublevel correlation spectroscopy (HYSCORE).

HYSCORE is a two-dimensional, four-pulse EPR technique that directly probes magnetic nuclei coupled to paramagnetic centers. This is an exceptionally sensitive technique that has the resolution necessary to distinguish and characterize individual nuclei. The magnitude of coupling for each magnetic nucleus is highly dependent on the distance separating it from the paramagnetic center and its position relative to the magnetic axis. Similar HYSCORE experiments have been performed on a variety of nonheme enzymes to provide structural details about the (substrate/NO)-bound active site geometry (12, 51, 52, 53, 54, 55, 56). Moreover, relative to Mössbauer spectroscopy, HYSCORE is much more sensitive to subtle structural perturbations in the outer coordination sphere of the Fe-site, allowing for a more robust validation of the optimized (3MPA/NO)-bound *Av*3MDO structure.

Figure 8 (panel *B*) shows a representative HYSCORE spectrum of the (3MPA/NO)-bound *Av*3MDO complex. This spectrum was measured at 175 mT near the low-field edge of the CW EPR spectrum (Fig. 8*A*) where there is no contribution from the small proportion of DNIC that appears near *g* = 2 (12, 38). The spectrum can be divided into two quadrants, the (−, +) quadrant, or the left half of the spectrum, and the (+, +) quadrant, or the right half of the spectrum. HYSCORE peaks appear in pairs that are reflected along the frequency diagonal at or near the nuclear Larmor frequency. The spectrum in Figure 8*B* encodes precise information about the nuclei coupled to the *S* = 3/2 center. The spectrum is comprised of multiple, overlapping peaks characteristic of coupled ^14^N and ^1^H from the coordinated histidine ligands, NO, 3MPA, and other nearby residues making up the second coordination sphere. The peaks in the (−, +) quadrant represent strongly coupled ^14^N nuclei; these peaks arise from nuclei where the hyperfine couplings are much greater than the ^14^N Larmor frequency at 175 mT, ∼0.54 MHz. These peaks are characteristic of what is observed for directly coordinated ^14^N from histidine ligands (53, 57). The (+, +) quadrant contains peak from weakly coupled nuclei, namely those where the ^14^N Larmor frequency is greater than the hyperfine coupling. Figure 8*B* shows several overlapping peaks in the (+, +) quadrant below ∼4 MHz; these peaks can be attributed to weakly coupled ^14^N. In general, the remote ^14^N of histidine ligands in metalloenzymes has a much weaker hyperfine coupling than the directly coordinated ^14^N (57). For example, the remote ^14^N in histidine residues coordinated to the diiron site of the hydroxylase component of methane monooxygenase was found to have an isotropic coupling of 0.8 MHz, whereas the coordinated nitrogen had a coupling of 13.0 MHz (58). Therefore, the peaks in the (+, +) quadrant below 4 MHz are likely the remote ^14^N on the three coordinated histidine ligands, although there could be a small contribution from second-sphere residues. In addition to ^14^N, the (+, +) quadrant reveals multiple pairs of coupled ^1^H peaks that are shifted from the ^1^H Larmor frequency by significant dipolar couplings. The most intense region of the ^1^H spectrum is an arc centered near (5, 12) MHz (ν_1_, ν_2_) that curves in toward the frequency diagonal. For simplicity, we refer to the coordinates of the peak that is on the high frequency side (larger ν_2_) of the diagonal since each peak is nearly symmetric about the diagonal. The arc consists of several underlying peaks with similar anisotropic hyperfine couplings. There are two additional sets of peaks with frequencies of (6, 7, 8, 9) MHz; these are more weakly coupled ^1^H as they appear closer to the ^1^H Larmor frequency along the diagonal.

**Figure 9:**
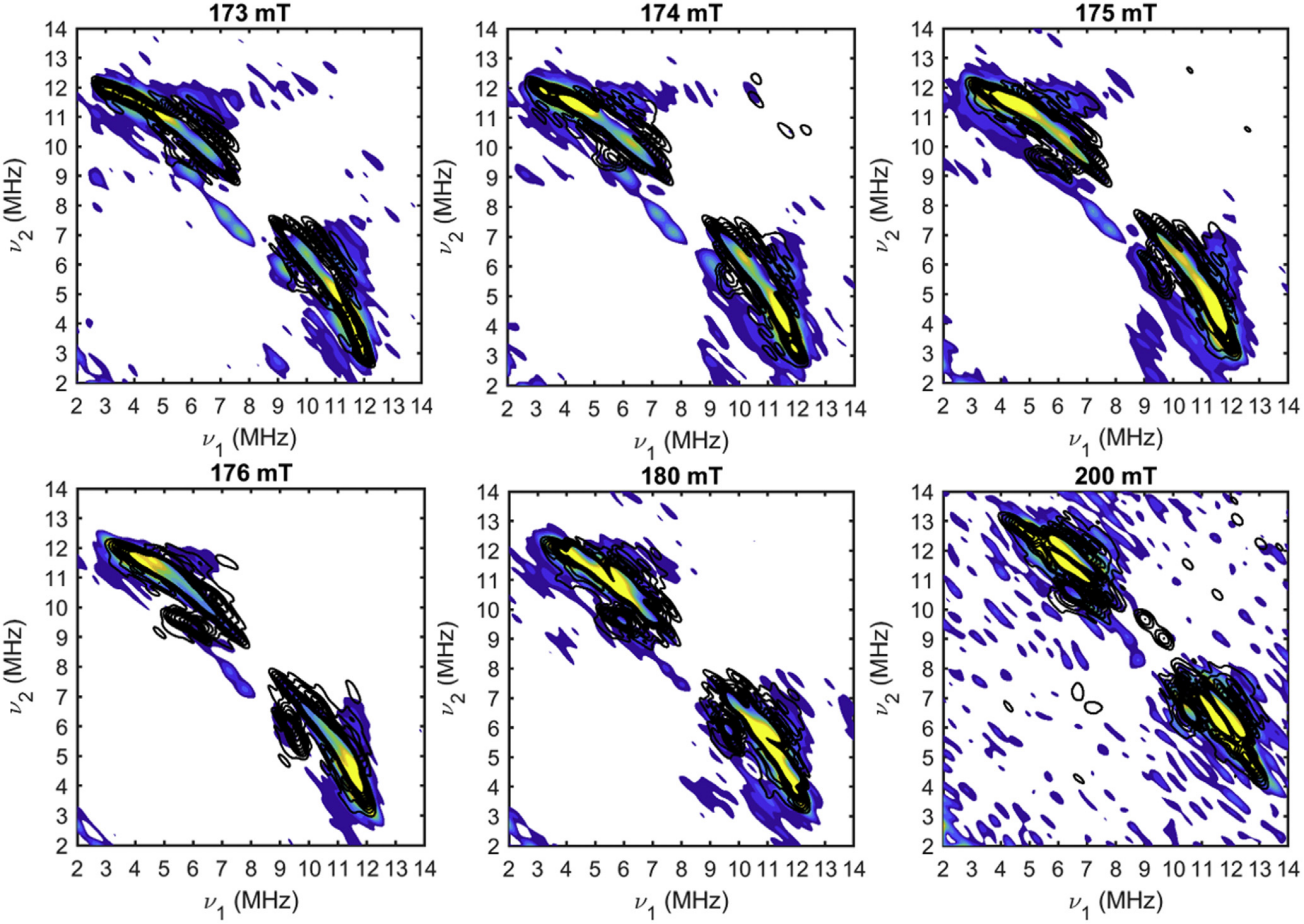
**Comparison of (3MPA/NO)-bound *Av*3MDO HYSCORE data (**^**1**^**H region) collected at six field positions (173–200 mT) to simulations using parameters from the DFT optimized model.** In each panel, experimental spectra are represented by the *color contours*, whereas simulations are overlaid in *black contours*. Data were collected from 173 to 200 mT using the following instrumental parameters: microwave frequency, 9.78 GHz; temperature, 5 K; pulse repetition rate, 1.25 kHz; τ, 120 ns. Additional instrumental parameters are described in detail in Experimental procedures.

In order to derive specific structural information from HYSCORE results, we carried out computational simulations for comparison to the experimental spectrum. In this case, the observed HYSCORE spectrum depends on several discrete factors. For an *I* = 1/2 nucleus like ^1^H, the peak positions are largely determined by the hyperfine coupling and the position of each nucleus with respect to the magnetic axis system. Beyond these parameters, additional complications arise for ^14^N (*I* = 1) quadrupole nuclei. For this reason, we chose to focus our attention solely on the ^1^H region of the spectrum. DFT calculations provide the geometry-optimized model from which distances and positions of each coupled ^1^H can be derived. As such, it is possible to simulate the HYSCORE spectrum by including all protons in the calculated model. However, the strongest couplings dominate the spectrum and the simulated intensity, which makes the intensity of weakly coupled protons difficult to distinguish. Accordingly, we focused on eight individual ^1^H with larger dipolar couplings (*T*_*total*_ derived from Equation 6, and listed in Table S5). These protons are located on the coordinated histidine residues and on C3 of 3MPA. Figure 9 shows HYSCORE spectra and simulations at magnetic fields spanning the low-field region of the EPR spectrum. The simulations take into account eight protons with axial hyperfine couplings, each with a hyperfine tensor and a set of angles that relate the Fe-^1^H vector and the magnetic axis system. Many of these peaks overlap due to the similarities in dipolar couplings. Figs. S7–S10 illustrate the individual contributions for each simulated ^1^H to the overall ^1^H HYSCORE spectrum. The hyperfine tensors and Euler angles, listed in Supporting Information (Table S5), were both derived directly from the DFT-optimized structure (see Experimental procedures for details).

The intensity of a ^1^H HYSCORE peak depends in part on measurement conditions, but the shape of the contours, or the peak “footprint,” shows the range of observed nuclear frequencies, which is largely independent of measurement conditions. Consequently, HYSCORE spectra are simulated by matching the peak locations and shapes rather than the intensities. The simulations in Figure 9, plotted as black contours, match the observed experimental contour shapes well. While the overlap of ^1^H peaks in the HYSCORE spectra limits our ability to optimize simulated contributions from individual ^1^H nuclei, the calculated parameters obtained from our computational model reasonably reproduce the entire ^1^H region of the observed spectra collected at multiple magnetic field positions. This later point is crucial as HYSCORE simulations are sensitive to changes in the hyperfine tensor and nuclear positions. To illustrate, Fig. S11 demonstrates how the simulated peaks for each ^1^H can shift considerably with relatively small changes in hyperfine coupling and/or its position relative to the magnetic axis. Accordingly, the fact that the simulations shown in Figure 9 reasonably overlay onto the experimental peaks across multiple magnetic fields confirms that the calculated model produces reasonable distances and locations for the ^1^H included in simulations. The protons in the optimized model that are not included in these simulations produce peaks that are too weak to resolve in the presence of the intense peaks from strongly coupled protons. The 3MPA protons on C2 fall into this category. We therefore performed deuterium exchange experiments as additional corroboration of bidentate 3MPA coordination. These experiments uniquely identify any solvent-based ligands that would be present in the case of monodentate 3MPA coordination. In principle, monodentate 3MPA coordination would be evident from the absence of strongly coupled ^1^H peaks in samples prepared in ^2^H_2_O-buffer. In order to test this, samples of (3MPA/NO)-bound *Av*MDO were prepared in ^2^H_2_O-buffer in order to observe Fe-coordinated solvent ligands and exchangeable protons *via* lost peaks in the ^1^H HYSCORE spectrum. However, as shown in Fig. S11, no such difference is observed in ^2^H_2_O samples. This observation is consistent with the absence of solvent-derived ligands directly coordinated to the Fe-site. Moreover, the observed nonexchangeable behavior is consistent with what is expected for each simulated group (Table S5).

Despite the inability to resolve the ^1^H peaks associated with 3MPA C2, a monodentate coordination of 3MPA can be reasonably ruled out as this would significantly alter the position and distance of the 3MPA C3 protons. Therefore, the ability to simulate the ^1^H HYSCORE spectra collected at multiple magnetic fields along with the absence of exchangeable Fe-bound solvent ligands provides ample validation of the optimized (3MPA/NO)-bound *Av*3MDO Fe-site model shown in Figure 7. Collectively, these results corroborate that the bidentate inhibitor coordination reflected in the *Av*3MDO-3HPA complex is retained in the 3MPA-bound ES-complex.

## Discussion

### Substrate coordination at the *Av*3MDO Fe-site

The structure of *Av*3MDO in complex with 3HPA and corroborating DFT computational modeling of the 3MPA-bound Fe-site are consistent with the bidentate substrate binding model proposed by Jameson and Karplus (9) where the substrate carboxylate group simultaneously interacts with iron as well as a nearby Arg168 residue. Interestingly, this Arg is nonhomologous to the Arg residue employed by CDO to bind the carboxylate group of CYS, demonstrating the phenomenon of active site plasticity within different lineages of thiol dioxygenases (59). Given the ambiguous oxidation state in the *Av*3MDO-3HPA complex, additional spectroscopic validation was performed to independently corroborate the nature of 3MPA coordination at the enzymatic Fe-site. Computationally predicted Mössbauer parameters (δ and ΔE_Q_) for the optimized (3MPA/NO)-bound *Av*3MDO site (Fig. 7) are consistent with reported experimental values, thereby providing support for a similar 3MPA coordination as present in the *Av*3MDO-3HPA complex. Moreover, the closeness of simulated HYSCORE ^1^H-couplings from coordinated His residues (His90, His92, and His142) and 3MPA C3 to observed spectra collected across multiple field positions makes a powerful argument for the validity of the optimized (3MPA/NO)-bound *Av*3MDO active site model. Further, since coordination of NO to the Fe-site defines the primary magnetic axis, ^1^H HYSCORE simulations also verify NO (and likely also dioxygen) binds to the axial Fe-site *trans* to His90. While ^1^H-peaks associated with 3MPA C2 are too weak to resolve, the absence of a difference in peaks between ^2^H_2_O/H_2_O HYSCORE spectra demonstrates that there are no solvent-derived ligands in the *Av*3MDO ES complex, further supporting the conclusion of bidentate coordination of the native 3MPA substrate at the enzymatic Fe-site. Collectively, the combined crystallographic, spectroscopic, and computational results provide an experimentally verified model for 3MPA binding within the *Av*3MDO active site resolving a debate in the literature regarding the denticity of substrate binding.

The chelate effect associated with bidentate 3MPA Fe coordination likely explains the ∼2000-fold increase in the *K*_*M*_ value obtained in CA-reactions [0.013 ± 0.005 *versus* 26.5 ± 5 mM] (14). As mentioned in the start of the text, we previously disregarded this possibility on the basis of our kinetic and CW EPR studies performed on *Av*3MDO using multiple thiol-bearing substrates (3MPA, CYS, CA, and ET) (14). Taken together with the results presented here, it is evident that while bidentate coordination of the native 3MPA substrate is favored, it is not obligatory for catalysis or O_2_ activation. For substrates lacking a carboxylate functional group, only coordination of a thiolate (presumably *trans* to H92) is required for gating O_2_ activation. This behavior is unlike mammalian CDO, which is highly specific for the L-isomer of CYS (35). Despite the ability to activate oxygen, *Av*3MDO reactions with non-carboxylate-bearing substrates are significantly uncoupled. For instance, in assays with 3MPA and CYS, the consumption of molecular oxygen is essentially stoichiometric with formation of the sulfinic acid. However, in reactions with CA, the coupling efficiency decreased by more than half [40 ± 9%] (14). Similarly, in reactions with the aromatic substrate 2-mercaptoanaline (2MA), O_2_ consumption was observed without formation of a sulfinic acid product. Instead, the majority products of this reaction appear to be hydrogen peroxide and 2MA disulfide (60, 61). By contrast, no oxygen consumption is observed in *Av*3MDO reactions with inhibitor (3HPA) in the absence of substrate. Similarly, no formation of iron-nitrosyl species is observed by EPR upon addition of nitric oxide to *Av*3MDO in the presence of excess 3HPA. Therefore, thiol Fe coordination is essential for initiating O_2_ activation; however, coordination of the substrate carboxylate appears to attenuate nonproductive “*off pathway*” reactions following binding of O_2_ to the ES complex.

### Orientation of hydrogen bonding network

The *Av*3MDO-3HPA structure reveals an apparent change in the directionality of the “SHY” proton relay network relative to the eukaryotic CYS-bound CDO. As shown in Fig. S6*A*, Trp77 of the mammalian CDO serves as an H-bond donor to Ser153 (W77 → S153). This dictates the direction of proton donation toward the Fe-site (*dashed blue lines*). By contrast, Glu105 of the *Av*3MDO-3HPA complex (Fig. S6*B*) is an H-bond acceptor, thus reversing (H157 → Ser155 → Glu105) hydrogen bond donation (*dashed red lines*). This leaves Tyr159 in a favorable position to donate a hydrogen atom to either His157 (*red*) or the axial Fe-bound chloride (*blue*). We have previously reported for both *Av*3MDO and *Mus musculus* CDO that perturbations within the “SHY” motif directly influence substrate specificity and denticity of Fe coordination, nitric oxide affinity, and the oxygen *K*_*M*_-value (12), as well as “*coupling*,” which is defined as the molar ratio of O_2_ consumed per sulfinic acid produced (35). Despite these observations, the functional role of this conserved proton relay network remains poorly understood. An obvious question is whether the orientation of the Tyr159 H-bond donation plays any role in gating O_2_ activation to coordination of the substrate at the Fe-site.

Energy surface scan calculations on the optimized *Av*3MDO-3HPA complex (Fig. S6*C*) predict that Tyr159 H-bond donation to the Fe-bound chloride ligand is more stable relative to the Tyr159 → His157 configuration. This conclusion is consistent with previously reported both EPR and Mössbauer studies verifying direct interaction between the SHY Tyr-residue and enzymatic Fe-site (12, 14, 31). However, the particular atom (N or O) receiving the hydrogen bond and how it would translate to catalytically relevant molecular oxygen remains unsettled. While attempts were made to directly observe and assign the Tyr159 hydroxyl proton by HYSCORE spectroscopy, the peaks overlapped with other weakly coupled ^1^H that had anisotropic hyperfine couplings of <1 MHz. These more distant ^1^H, which include the ^1^H on the remote nitrogen of the three histidine residues and the ^1^H on Arg168, overlap near the matrix ^1^H peak that occurs at the nuclear Larmor frequency along the diagonal (Shown in Fig. S13). Accordingly, the direction of the Tyr159 H-bond donation could not be corroborated spectroscopically.

### Implications of the closed active site cavity observed for *Av*3MDO

A notable finding from our structural analysis of *Av*3MDO was its lack of a patent tunnel for substrate diffusion to the active site. This observation was consist across all crystallographically independent protomers of both *Av*3MDO crystal forms. This structural uniformity suggests that the closed conformation could be adopted in the solution form of the enzyme, although the fact that it is observed in both the presence and absence of 3HPA seems to rule out the possibility that active site closure could be triggered by substrate binding. Such a uniformly occluded conformation has not, to our knowledge, been observed for other thiol dioxygenases, although two of the four subunits of the *Pa*3MDO crystal structure do appear to be occluded. Clearly, the active site must communicate with the bulk solvent to allow organic substrate entry, and the tunnel described in Figure 4 is the best candidate for such a role given its appropriate size and orientation with respect to the iron center. Discerning the relevance of the closed conformation observed for *Av*3MDO to the thiol dioxygenase catalytic cycle will require further research.

## Experimental procedures

### Protein expression and purification

A detailed description for the expression and purification of this enzyme has been reported elsewhere (12, 14). Briefly, the *Av*3MDO expression vector was transformed into chemically competent BL21(DE3) *E. coli* (Novagen Cat. no. 70236-4) by heat shock and grown overnight at 37 °C on a LB-agar plate containing 100 mg/l ampicillin (Amp). A single colony was selected for training on antibiotic in liquid LB media prior to inoculation of the 10-L BF-110 fermenter (New Brunswick Scientific) at 37 °C. Cell growth was monitored by optical density at 600 nm (OD 600). Induction was initiated by adding 1.0 g isopropyl β-*D*-1-thiogalactopyranoside (IPTG), 78 mg ferrous ammonium sulfate, and 20 g casamino acids at an OD600 value of ∼4. During induction, the temperature of the bioreactor was decreased from 37 to 25 °C and agitation was set to maintain an oxygen concentration of 20% relative to air-saturated media. Four hours postinduction, cells were harvested and pelleted by centrifugation (Beckman-Coulter Avanti J-E, JA 10.5 rotor). The resulting cell paste was stored at −80 °C.

In a typical purification, ∼20 g frozen cell paste was added to extraction buffer [20 mM 4-(2-hydroxyethyl)-1-piperazineethanesulfonic acid (HEPES), 50 mM NaCl, pH 8.0]. Lysozyme, ribonuclease, and deoxyribonuclease were added to the slurry for a final concentration of 10 μg/ml each and stirred slowly on ice prior to pulse sonication (Bronson Digital 250/450). The insoluble debris was removed from the cell-free extract by centrifugation at 48,000*g* for 1 h at 4 °C. The supernatant was diluted 1:1 with extraction buffer and then loaded onto a DEAE sepharose fast flow anion exchange column (GE Life Sciences #17070901) pre-equilibrated with 20 mM HEPES, 50 mM NaCl, pH 8.0. The column was washed with three column volumes of extraction buffer prior to elution in a linear NaCl gradient (50 mM–350 mM).

Fractions were collected overnight and pooled based on enzymatic activity and/or SDS PAGE as described elsewhere (12). The pooled enzyme solution was concentrated to ∼50 ml using an Amicon stir cell and YM-10 ultrafiltration membrane prior to thrombin protease (Biopharma Laboratories) cleavage to remove C-terminal His-tag from the expressed enzyme. Following overnight cleavage at 4 °C, the enzyme was dialyzed against 4 l of 20 mM HEPES, 20 mM NaCl at pH 8.0 to decrease the salt content. A second DEAE column separation in a linear gradient (50–250 mM NaCl) was used to separate the free (His)_6_-tag from the purified enzyme. Precrystallization desalting was performed by Sephadex G25 column equilibrated with 20 mM HEPES, 10 mM NaCl at pH 8.0 prior to concentrating drop freezing in liquid nitrogen and storage at −80 °C. Iron content was quantified spectrophotometrically for both ferric and ferrous concentration using 2,4,6-tripyridyl-s-triazine (TPTZ) in a method previously described (14, 38).

### Enzyme assays

The rate of dioxygen consumption in activity assays was determined polarographically using a standard Clark electrode (Hansatech Instruments, Norfolk, England) in a jacketed 2.5 ml cell. Reaction temperatures were fixed at 25 °C ± 1 °C using a 5 l circulating water bath (Grant Instruments) and O_2_-electrode calibration was performed as described elsewhere (12, 31, 62). For all 3MPA concentrations [7–1000 μM], reactions were initiated by addition of 5.0 μM Fe(II)-*Av3*MDO. Steady-state kinetic parameters for the enzyme used in experiments were consistent with previously published values at 25 °C [*k*_*cat*_, 1.0 ± 0.1 s^−1^; *K*_*M*_, 13 ± 5 μM; *k*_*cat*_/*K*_*M*_, 72,000 ± 9200 M^−1^ s^−1^] (14).

### Data analysis

Inhibition kinetic results were fit globally using the *Enzyme Kinetics Add-On* module of SigmaPlot ver. 14.0 (Systat Software Inc). In the presence of a competitive inhibitor, the Michaelis–Menten and Lineweaver–Burk equations take the form shown in Equations 1 and 2, respectively. From the analysis, kinetic parameters (*V*_*max*_, *K*_*M*_, and *K*_*I*_) as well as the error associated with each value were determined by nonlinear regression.

(1) $$v_{0}\;=\;\frac{V_{max}\left[S\right]}{K_{M}\cdot \left(1\;+\;\frac{\left[I\right]}{K_{I}}\right)\;+\;\left[S\right]}$$

(2) $$\frac{1}{v_{0}}\;=\;\frac{K_{M}\cdot \left(1\;+\;\frac{\left[I\right]}{K_{I}}\right)}{V_{max}}\cdot \frac{1}{\left[S\right]}\;+\;\frac{1}{V_{max}}$$

### *Av*3MDO crystallization

*Av*3MDO crystallization conditions were identified through sparse matrix trials using a number of commercially available screens. The initial hit (crystal form A) was obtained in condition 2-42 of the MIDAS screen (Molecular Dimensions), which consists of 100 mM MES pH 6, 30% w/v poly(acrylic acid sodium salt) 5100, and 10% v/v ethanol. Two microliters of *Av*3MDO at a concentration of 40 mg/ml was mixed with 2 μl of the crystallization cocktail on a siliconized coverslip (Hampton Research), and the resulting drop was incubated over 500 μl of the same crystallization cocktail in a sealed chamber at 22 °C. Thin needle-shaped crystals with a tendency to grow in clusters were observed after a week of incubation. These initial crystals were replicated with independently prepared solutions and further optimized through streak seeding and inclusion of 50 mM MgCl_2_ in the crystallization cocktail. The optimized crystals grew as thin rods over the course of a week with approximate final dimensions of 50 × 50 × (100–800) μm. Most crystals exhibited a splitting defect along their long axis. The crystals were harvested directly from the mother liquor using elliptical Microloops (Mitegen) and flash frozen in liquid nitrogen.

To obtain an alternative *Av*3MDO crystal form, *Av*3MDO was further purified by anion exchange and gel filtration chromatography in the absence of reducing agents and then concentrated to 40 mg/ml. A second crystallization hit (crystal form B) was obtained in condition E4 of the MidasPLUS screen (MD1-107, Molecular Dimensions), which consists of 100 mM HEPES pH 7, 40% v/v pentaerythritol propoxylate (5/4 PO/OH), and 0.2 M sodium thiocyanate. Two microliters of *Av*3MDO at a concentration of 40 mg/ml was mixed with 2 μl of the crystallization cocktail on a siliconized coverslip (Hampton Research), and the resulting drop was incubated over 500 μl of the same crystallization cocktail in a sealed chamber at 22 °C. Yellowish, rod-shaped crystals with “feather-duster” growth defects on each end were observed after 2 to 5 days of incubation. The growth defects were minimized by changing the HEPES, pentaerythritol propoxylate, and sodium thiocyanate concentrations to 50 mM, 44%, and 0.6 M, respectively. Mature (150 × 150 × 600 μm) single crystals were harvested directly from the mother liquor using Microloops (Mitegen) and flash frozen in liquid nitrogen.

### X-ray diffraction data collection, processing, and analysis

*Av*3MDO crystal diffraction data were collected using beamlines 17-ID-2 (FMX) at the National Synchrotron Light Source (NSLS)-II, beamline 12-2 at the Stanford Synchrotron Light Source (SSRL) and beamline 24-ID-E (NE-CAT) at the Advanced Photon Source (APS). All form A *Av*3MDO crystals examined were twinned to various degrees as described in more detail below. The microfocusing capability of the FMX 17-ID-2 beamline was used to collect data from small crystal volumes to minimize contributions from twin-related domains. Initial crystals diffracted to ∼2.7 Å resolution and the diffraction was somewhat anisotropic, being strongest along c∗. Weak diffuse scatter was observed between reflections in h-k planes of reciprocal space. Data were indexed, integrated, and scaled using XDS (63). The data were initially processed in space group *P*622 and examination of axial reflections along c∗ indicated the presence of a 6_2_ or 6_4_ screw axis. Analysis of the intensities with the L-test (64), as implemented in *phenix.xtriage* (65), provided strong indication that the data were twinned. The data were therefore reprocessed in space groups *P*321 and *P*3 with similar *R*_merge_ values for each. H-tests (66) for the four possible unique twin operators in the latter point group indicated that the crystals were tetartohedrally twinned. Crystal streak seeding and the inclusion of MgCl_2_ in the crystallization cocktail substantially reduced crystal twinning and improved the data resolution to ∼2.25 Å. The optimized crystals remained partially merohedrally twinned with respect to operator (k, h, −l). The best crystals of crystal form B diffracted X-rays to ∼2.9 Å resolution. Data were processed in space group *P*6(_1,5_)22. While no twinning was detected in this crystal form, the native Patterson map suggested the presence of pseudotranslational symmetry. X-ray data collection statistics are shown in Table S1.

### Structure refinement and analysis

Structure solution for crystal form A was carried out by molecular replacement in *Phaser* (67) using the coordinates of a related 3MDO protein (5) as a search model (PDB accession code 4TLF). Structure solution was attempted, unsuccessfully, in space groups *P*6(_2, 4_)22 before it was realized the data were twinned. Space groups *P*3(_1, 2_)12 were excluded from further consideration owing to poor merging statistics. MR trials in space groups *P*3(_1, 2_)21 led to a clear solution in space group *P*3_1_21 with four molecules (a pair of dimers) per asymmetric unit and a solvent content of 64%. Amino acid substitutions and model adjustments were made using *Coot* (68) and the model was refined using *Refmac* (69). After several rounds of model building and refinement, *R*_free_ plateaued at ∼37%. At this point, a majority of the strongest residual electron density was found within a large solvent channel, but it was largely uninterpretable. The possibility of rotational pseudosymmetry causing an elevation in the apparent crystal symmetry was considered and the partially refined *Av*3MDO coordinates were used as a search model for molecular replacement in space group *P*3_1_. *Phaser* located 12 molecules in the asymmetric unit giving a solvent content of 47%. Refinement of this new model against the *P*3_1_ processed data (with *R*_free_ reflections transferred from the original data set after symmetry expansion to *P*3_1_) resulted in an immediate reduction of *R*_free_ to ∼28% and elimination of the residual density features noted above. RvR analysis (70) of the *P*3_1_ data and calculated amplitudes as implemented in *phenix.xtriage* (65) revealed the presence of twofold rotational pseudosymmetry along the a and b axes together with partial merohedral twinning (k, h, −l with α ∼ 0.34). These features were also evident from inspection of Patterson self-rotation plots, which showed strong chi = 180° peaks along the “a” and “b” axes. Together, these features accounted for the similar *R*_merge_ values for space groups *P*3_1_21 and *P*3_1_. The space group assignment was further validated using Zanuda (71) and labelit.check_pdb symmetry (72) as well as by manual inspection of the crystal packing environment for each of the monomers in the asymmetric unit. A monomer from the *P*3_1_ model was then used as a model to solve crystal form B by molecular replacement in space group *P*6_1_22 with four monomers in the asymmetric unit.

Refinement of both models was completed by alternating reciprocal space refinement in *Refmac*, using the amplitude-based twin refinement option in the case of crystal form A, and manual model improvements in *Coot*. Coordinate and dictionary files for the test ligands were generated using the GRADE server (Global Phasing LTD). In the case of crystal form A, inspection of residual density near the iron center revealed a large peak in the axial position and an elongated, continuous feature in the equatorial positions, indicative of a diffusible ligand set different from the three aquo complex modeled in the structure of *Pa*3MDO (Fig. S1*A*) (11). Indeed, placement of three water molecules poorly explained the residual density as shown in Fig. S1*B*. The density *trans* to His142 was flat and appeared to make a simultaneous interaction with Arg168, suggesting it could represent a carboxylate-containing ligand. Modeling of a bicarbonate at this position resulted in an excellent fit to the electron density map (Fig. S1*C*). However, the bicarbonate-H_2_O-H_2_O model resulted in residual positive density features at the axial position *trans* to His 90 as well as between bicarbonate and the water ligand *trans* to His92 in addition to an unacceptably close interaction (2.1 Å) between the nonaxial water and bicarbonate ligands (Fig. S1*C*). Replacing the axial water with a chloride ion, which was present in the crystal mother liquor at ∼100 mM concentration, fully quenched the residual density with a reasonable refined *B*-factor (Fig. S1*D*). Chloride binding at the equivalent site in CDO was previously reported (27, 41), which further supports the plausibility of our assignment. The difference map analysis described above suggested that the equatorial features represented a single carboxylate-containing compound. Since known components of the crystal mother liquor could not account for the electron density, we further considered possible contaminants that the density could represent. 3-hydroxypropionic acid is a known precursor and contaminant of the polyacrylate used for *Av*3MDO crystallization, which we confirmed by mass spectrometry, and was considered the most likely source of the electron density feature (73, 74). Additionally, 3HPA is a close analog of the 3MPA substrate of *Av*3MDO. Placement of 3HPA at this position completely quenched the residual density with refined *B*-factors closely matching those of iron and its other ligands (Fig. S1*E* and Table S1). We also attempted to model 3MPA into the density, but observed a residual density hole near the sulfur atom, which also exhibited an elevated *B*-factor relative to the remainder of the molecule (Fig. S1*F*).

The initial difference maps for the crystal form B model also revealed density in both the equatorial and axial positions. The equatorial features were interpreted as two coordinated water molecules while the axial density was elongated and not adequately satisfied by modeling either a single water or chloride ligand. The elongated density instead strongly suggested a bound thiocyanate ligand, which was modeled with its nitrogen atom directly coordinating the iron ion based on difference map analysis and refined *B*-factors computed with the ligand modeled in either orientation.

Structure validation was carried out with the Molprobity (75) and wwPDB (76) web servers. The final model statistics are shown in Table S1. The coordinates and structure factor amplitudes have been deposited in the Protein Data Bank under accession code 6XB9 (crystal form A) and 7KOV (crystal form B).

### Mass spectrometry

Sodium polyacrylate 5100 solution (Hampton Research) was diluted to 10% (v/v) with Millipore water. In total, 100 μl of the resulting solution was mixed with 900 μl of HPLC-grade acetone to precipitate the acrylate polymer and the mixture was centrifuged at 17,000*g* for 15 min. The supernatant (10 μl) was diluted with 50% HPLC-grade methanol (90 μl), and 10 μl of the resulting solution was analyzed with LXQ Mass spectrometer (Thermo Scientific) coupled with an Ultimate 3000 HPLC system. 3HPA standard (Sigma-Aldrich) was prepared in an identical manner. Chromatography was performed with a Poroshell 120 EC C18 Column (2.7 μm, 4.6 mm × 50 mm, Agilent Technologies) using a gradient mobile phase of acetonitrile/2-propanol (1/1, v/v) in water from 20% to 98% over 20 min. The flow rate was 0.6 ml/min. Ions were detected in negative mode with a normalized collision energy of 35%.

### Pulsed EPR measurements

HYSCORE measurements were made using an ELEXSYS E680 EPR spectrometer (Bruker-Biospin) equipped with a Bruker Flexline ER 4118 CF cryostat and an ER 4118X-MD4 ENDOR resonator. Measurements were made at 5 K with a nominal EPR frequency of 9.78 GHz and used a four-pulse sequence, π/2−τ−π/2−t1−π−t2−π/2−τ−echo. This sequence was repeated at a rate of 1.25 kHz with values of 16 ns and 32 ns for the π/2 and π pulses, respectively. The times t1 and t2 were varied independently from 48 ns to 3096 ns in increments of 24 ns for a total of 128 points in each dimension. The delay time τ was set to 120 ns to maximize resolution in the ^1^H region of the spectrum. HYSCORE spectra were processed using custom scripts in MATLAB (Mathworks, R2020a). Briefly, the complex raw data was phased to minimize the imaginary component, and the background decay was subtracted in each dimension by removing a second-degree polynomial. Then, a diagonal Blackman apodization function was applied to minimize noise at larger values of t1 and t2. The data were then zero-filled in both directions to 1024 points before calculating the two-dimensional Fourier transform. The absolute value of the real part of the Fourier transform is displayed. HYSCORE peaks are symmetric about the frequency diagonal; however, several factors, including interference from strong nuclear modulation, can affect the intensity of each peak. As a result, real peaks are roughly symmetric about the frequency diagonal, but they can have minor intensity variations. Noise, on the other hand, is not symmetric about the diagonal. Therefore, the spectra were left unsymmetrized in order to best distinguish between signal and noise. Spectra were simulated using the “saffron” function in EasySpin, a comprehensive EPR toolbox in MATLAB (77).

HYSCORE simulations on the ^1^H region are based off the spin Hamiltonian

(3) $$\hat{H}\;=\;-\gamma_{H}\hat{I}\cdot B\;+\;\hat{S}\cdot \tilde{A}\cdot \hat{I}$$

where $-\gamma_{H}$ is the proton gyromagnetic ratio, $\hat{S}$ and $\hat{I}$ are the electron and nuclear spin operators, *B* is the magnetic field vector, and $\tilde{A}$ is the hyperfine coupling tensor. The proton couplings were modeled using an axial dipolar tensor,

(4) A=[−T,−T,(2∗T)]

where *T* is the anisotropic contribution of the hyperfine interaction. The tensor was considered purely dipolar due to the distance of the coupled ^1^H, which is consistent with other HYSCORE analyses of {FeNO}^7^ centers (52, 53, 54). The dipolar contribution, *T*, was calculated using the geometry-optimized DFT structure of 3MPA-bound *Av*3MDO treated with NO and spin-projection factors that take into account the electronic structure of the {FeNO}^7^ center (52, 54). In brief, distances between the Fe, the N, and the O of NO and each ^1^H were used to calculate a *T*_*Fe*_, *T*_*N*_, and *T*_*O*_ according to the point-dipole approximation.

(5) $$T\;=\;\left(\frac{\mu_{0}}{4\pi }\right)\frac{g_{e}g_{n\beta_{e}\beta_{n}}}{hr^{3}},$$

where $\mu_{0}$ is the permittivity of free space, *g*_*e*_ is the electronic *g-value*, *g*_*n*_ is the nuclear *g-value*, *β*_*e*_is the electronic Bohr magneton, *β*_*n*_ is the nuclear Bohr magneton, *h* is Planck’s constant, and *r* is the distance from the proton to the Fe, N, or O of the {FeNO}^7^ center. The dipolar contributions were then summed using spin projection factors.

(6) $$T_{total}\;=\;\frac{7}{5}\left(T_{Fe}\right)-\frac{1}{5}\left(T_{N}\;+\;T_{O}\right)$$

where *T*_*Fe*_, *T*_*N*_, and *T*_*O*_ are the dipolar couplings resulting from the point-dipole approximation using the distance between the ^1^H and the Fe, N, and O of NO, respectively. The couplings for the ^1^H on His90 and His142 were varied by ±0.1 Å, which is the estimate of the precision of the atomic locations in the crystal structure. Equations 5 and 6 were used to transform the ±0.1 Å variation into the corresponding value in MHz. The other ^1^H couplings were left unvaried. For each ^1^H, the dipolar coupling between the ^1^H and the Fe dominates Equation 6; therefore, the vector connecting each ^1^H and Fe is a reasonable approximation of the hyperfine interaction (52). In addition to the hyperfine coupling, HYSCORE simulations take into account the transformation of the hyperfine tensor into the magnetic axis system. This transformation is defined by a set of Euler angles (α, β, γ) in EasySpin that follow the zy′z′ convention. In the case of an axial hyperfine interaction, only two angles are necessary. As with other {FeNO}^7^ centers, the principal axis of the zero field splitting (zfs) and the molecular z axis are nearly coincident (52, 53). As a result, these Euler rotations can be approximated using polar angles φ and ɵ, where φ is taken as the angle between the proton and the x-z plane, and ɵ defines the deviation from the z-axis defined by the Fe-NO bond. An illustration below Table S3 shows how these angles relate to structure for a ^1^H on 3MPA. All angles were measured based on the geometry-optimized structure using Chimera 1.14 (UCSF) (78). While simulations were not very sensitive to the value of φ, the value of ɵ was varied for His90 and His142 by <10% for the best fit.

### Computational modeling

All calculations were performed using Orca version 4.2 (79). Starting coordinates for geometry optimizations were extrapolated from the crystal structure presented in this work and the crystal structure of *Pa*3MDO, PDB 4TLF. Optimizations were done by capping the alpha-carbons of each residue with a methyl group. Histidines directly coordinated to the iron were capped at the beta-carbon. Preliminary optimizations were done by optimizing only hydrogens and constraining the dihedral angles of capped methyl group hydrogens relative to the constrained main group atoms. These methyl groups were constrained in all geometry optimizations. Histidines coordinated to iron were protonated at the δ-position. Geometry optimizations utilized two methods. Most optimizations used the BP’86 functional with the Ahlrichs def2-tzvp basis set on iron and directly coordinated atoms and the def2-svp basis set on all other atoms (80, 81, 82). This method has been shown to work well for transition metal complexes (83). The nitroxide containing complexes used the B3LYP functional with the same basis set allocation as described earlier. B3LYP was chosen among other tested functionals (*e.g.*, BP’86, TPSS, PBE, and PBE0), as it was the only functional that could reproduce the expected antiferromagnetically coupled *S* = 3/2 {FeNO}^7^ electronic structure with *S* = 5/2 for iron and *S* = −1 on the nitrosyl ligand. Additionally, the iron nitrosyl complexes used the “broken-symmetry” approach where the wavefunction was calculated as a high spin *S* = 7/2 system before flipping the sign of the spin on the nitrosyl atoms to converge to an *S* = 3/2 system (84). All calculations utilized Grimme’s D3 dispersion correction, a CPCM solvent model with ε = 4 to emulate a protein environment, and either the resolution of identity (RI) for BP’86 or resolution of identity and chain of sphere (RIJCOSX) approximation for B3LYP with def2/J auxiliary basis sets (85, 86, 87, 88).

Mössbauer parameters were calculated using the B3LYP functional with the CP(PPP) basis set for iron and def2-TZVP on all other atoms. Orca specific settings featured the radial integration accuracy raised to 7 for the iron atom and a TightSCF convergence threshold with Grid5 and FinalGrid7 for the whole structure. Quadrupole splitting (ΔE_Q_) and η were calculated directly in Orca using the electric field gradient on the iron nucleus. The isomer shift (δ) was determined from the method described by Remolt *et al.* (89) using the s electron density at the iron nucleus.

EPR parameters were calculated with the same method described for Mössbauer parameter, with the exception of the IGLO-II basis set used for sulfur and EPR-II used for all other atoms (CP(PPP) was still used for iron). The spin–orbit coupling part of the zero field splitting tensor was calculated with the coupled-perturbed method and the spin–spin part was calculated using the spin density of “UNO” orbitals (84). Isotropic and dipolar contributions of hyperfine coupling were calculated for the nitrogens of H90, H92, H142, and Arg168 and the hydrogens of H90, H92, H142, Arg168, 3MPA, and the phenol hydrogen of Tyr159.

## Data availability

X-ray crystal structure data that support the findings of this study have been deposited in the Worldwide Protein Data Bank. The PDB accession codes for the *Av*3MDO complex with 3HPA and thiocyanate are 6XB9 and 7KOV, respectively.

## Supporting information

This article contains supporting information (12, 13, 14, 31, 40, 47, 48, 49, 61, 91, 92).

## Conflict of interest

The authors declare that they have no conflicts of interest with the contents of this article.
