# Supplementary material for: Structure of 3-mercaptopropionic acid dioxygenase with a substrate analog reveals bidentate substrate binding at the iron center
Source: J Biol Chem. 2021 Mar 1;296:100492. doi: 10.1016/j.jbc.2021.100492 (PMC8050391; doi:10.1016/j.jbc.2021.100492)
Supplement: Supplemental Figures S1–S13 and Tables S1–S5 [file mmc1.pdf]

Structure of 3-mercaptopropionic acid dioxygenase with a substrate analog reveals bidentate substrate binding at the iron center

Nicholas J. York[1], Molly M. Lockart[1], Sinjinee Sardar[2], Nimesh Khadka[3], Wuxian Shi[4], Ronald E. Stenkamp[5], Jianye Zhang[6], Philip D. Kiser*[,6,7,8], and Brad S. Pierce*[1]

[1] Department of Chemistry & Biochemistry, University of Alabama, 250 Hackberry Lane, Tuscaloosa, Alabama 35487, United States

[2] Department of Chemistry & Biochemistry, The University of Texas at Arlington, 700 Planetarium Place, Arlington, Texas 76019, United States

[3] Department of Pharmacology, Case Western Reserve University, 10900 Euclid Avenue, Cleveland, OH 44106, United States

[4] National Synchrotron Light Source-II, Brookhaven National Laboratory, Upton, NY, United States

[5] Departments of Biological Structure and Biochemistry, University of Washington, 1705 NE Pacific Street, Seattle, WA 98195, United States

[6] Department of Ophthalmology, School of Medicine, University of California, Irvine, 837 Health Sciences Road, Irvine, CA 92697, United States

[7] Department of Physiology & Biophysics, School of Medicine, University of California, Irvine, 837 Health Sciences Road, Irvine, CA 92697, United States

[8] Research Service, VA Long Beach Healthcare System, 5901 E 7th St, Long Beach, CA 90822, United States

Running title: *Mode of substrate binding in a thiol dioxygenase*

* To whom correspondence should be addressed: Department of Chemistry and Biochemistry, University of Alabama Tuscaloosa, Alabama 35487, USA, Telephone: (205)348-8445. E-mail: bspierce1@ua.edu
Department of Physiology & Biophysics, University of California Irvine, California 92617, USA, Telephone: (949)824-6954. Email: pkiser@uci.edu

Keywords: Thiol dioxygenase, non-heme iron, oxygenase, competitive inhibition, structure, DFT, computational modeling, iron-nitrosyl, FT-EPR, HYSCORE

SUPPLEMENTAL TABLES

Table S1. X-ray diffraction data collection and structure refinement statistics

Data collection and processing[1]		
Crystal	3-HPA complex (crystal form A)	Thiocyanate complex (crystal form B)
X-ray source	NSLS-II (FMX 17-ID-2)	APS (NE-CAT 24-ID-E)
Wavelength (Å)	0.979339	0.979180
Space group	$P3_1$	$P6_122$
Unit cell lengths (Å)	a = 178.22, c = 75.92	a = 102.18, c = 301.93
Resolution (Å)†	50 – 2.25 (2.39 – 2.25)	50 – 2.95 (3.13 – 2.95)
Unique reflections	127,973 (20,673)	20,505 (3,185)
Multiplicity	4.4 (4.4)	12.9 (13.4)
Completeness (%)	99.9 (99.8)	99.8 (99.2)
$<I/\sigma I>$	6.35 (0.78)	12.9 (0.98)
$R_{merge}I$ (%)	15.0 (164.2)	18.5 (288.3)
$CC_{1/2}$ (%)	99.3 (48.4)	99.9 (46.8)
Wilson *B* factor ($Å^2$)	55	86
Refinement		
Resolution (Å)	46.26 – 2.25	49.88 – 2.95
No reflections‡	121,709 (6,243)	19,476 (1,026)
R_{work}/R_{free} (%)†	19.6 (22.6)	21.4 (24.1)
Twin law (α)∀	k, h, -l (0.34)	N/A
No. atoms	18,870	6,166
Protein	18,374	6,124
Iron	12	4

3-Hydropropionic acid	72	---
Chloride/thiocyanate	13 (Cl^-)	12 (SCN^-)
Water	395	24
<*B*-factor> (Å^2)	57	105
Protein	57	105
Iron	52	86
3-Hydroxypropionic acid	53	---
Chloride/thiocyanate	63	122
Water	50	81
RMS deviations		
Bond lengths (Å)	0.006	0.003
Bond angles (°)	1.27	1.22
Ramachandran plot (% favored/outliers)*	96.4/0	94.0/0
Molprobity score (%)	100	100
PDB accession code	6XB9	7KOV

[1]Each data set was collected from a single crystal

[†]Values in parentheses are for the highest resolution shell of data

[‡]Values in parentheses are the number of reflections used for cross-validation

[▿]Twin fraction was refined in REFMAC based on the observed and calculated amplitudes

[*]Evaluated using Molprobity

Table S2. Comparison of *Av*3MDO bond distances with other thiolate-bound non-heme iron complexes. Distances are shown for the iron to bound thiolate (Fe-S), either bidentate bound substrate carboxylate, amine, or equivalent (Fe-O or Fe-N_{amine}), and average iron to histidine or equivalent nitrogen. All units are in Ångströms.

	Fe-S	Fe-O	Fe-N_{amine}	Fe-$N_{his\ ave}$	ref
*Av*MDO-**3HPA** complex[a]	2.16[b]	2.17	-	2.16	This work
**Fe^{II} structures**					
*Av*MDO **3HPA**$^{1-}$-bound[c]	2.26[b]	2.25	-	2.15	This work
*Av*MDO **3HPA**$^{2-}$-bound[c]	1.91[b]	2.55[d]	-	2.17	This work
*Av*MDO **3MPA**-bound[c]	2.33	2.51[d]	-	2.19	This work
*Rn*CDO cysteine-bound[a]	2.35	-	2.35	2.17	(1)
[Fe^{II}(2-MTS)(^{Ph2}TIP)]BPh_4[a]	2.32	2.12	-	2.16	(2)
[Fe^{II}(CysOEt)(^{Ph2}TIP)]BPh_4[a]	2.31	-	2.26	2.17	(3)
[Fe^{II}(Me_3TACN)(abt)(OTf)][a]	2.44	-	2.23	2.25	(4)
[Fe^{II}(Me_3TACN)(abt^{CF3})(OTf)][a]	2.43	-	2.24	2.24	(4)
[Fe^{II}(iPr_3TACN)(abt)](OTf)[a]	2.36	-	2.26	2.20	(4)
[Fe^{II}(iPr_3TACN)(abt^{CF3})](OTf)[a]	2.38	-	2.27	2.20	(4)
**Fe^{III} structures**					
*Av*3MDO **3HPA**$^{1-}$-bound[c]	2.23[b]	1.97	-	2.13	This work
*Av*3MDO **3HPA**$^{2-}$-bound[c]	1.87[b]	2.14	-	2.18	This work
*Av*3MDO **3MPA**-bound[c]	2.32	2.08	-	2.20	This work
[Fe^{III}(2-MTS)(^{Ph2}TIP)]-CN[c]	2.20	1.96	-	2.17	(2)
[Fe^{II}(CysOEt)(^{Ph2}TIP)]-CN[c]	2.18	-	2.05	2.17	(2)
(L-Cys/CN)-CDO cross-linked[c]	2.28	-	2.09	2.07	(5)
(L-Cys/CN)-CDO non-cross-linked[c]	2.26	-	2.11	2.05	(5)

[a] Distances obtained from crystal structure. [b] 3-oxide of **3HPA** instead of sulfur. [c] Distances obtained from computational methods. [d] To some extent, the longer Fe-$O_{(carboxylate)}$ distance of 2.51 - 2.55 Å can be attributed to the is likely attributed to electrostatic interaction of the anionic carboxylate group with adjacent Arg168. However, a larger issue is the simultaneously accommodation of three negative charges [Cl^- and **3HPA**$^{2-}$ (or **3MPA**$^{2-}$)] directly coordinated to the Fe-site resulting in a monoanionic active site complex, ([$Fe^{II}$$(His)_3$$Cl^-$(**X**$^{2-}$)]$^{1-}$, **X** = **3HPA**$^{2-}$ or **3MPA**$^{2-}$). This suggests that chloride is an unfavorable ligand for the catalytically relevant **3MPA**-bound Av3MDO Fe(II)-site.

Table S3. Geometric properties of the DFT optimized (**3MPA**/NO)-Fe(II) *Av*3MDO models. Three structures were optimized differing in the hydrogen bond donation of the Tyr159 hydroxyl group.

	Tyr-OH → $N_{(NO)}$	Tyr-OH → $O_{(NO)}$	Tyr-OH → Nδ-His157
Distance (Å)			
Fe-S	2.38	2.40	2.41
Fe-$O_{(carb)}$	2.08	2.08	2.11
Fe-$N_{(NO)}$	1.89	1.84	1.82
N-O	1.18	1.18	1.17
Fe-$His_{(Ave)}$	2.21	2.23	2.24
Fe-H90	2.19	2.23	2.19
Fe-H92	2.24	2.26	2.29
Fe-H142	2.21	2.20	2.23
Tyr-OH-*X*[a]	2.11	1.98	1.78
Angles ∠ (°)			
Fe-S-C_{α}	95.7	97.8	94.0
Fe-N-O	138.7	145.7	151.0
H90-Fe-$N_{(NO)}$	170.0	176.8	168.9
H92-Fe-S	174.8	175.2	173.9
$O_{(carb)}$-Fe-S	95.0	93.8	95.3
**3MPA** C_1-C_2-C_3	120.4	120.1	118.8

[a] *X* refers to the hydrogen binding partner of the Tyr157 hydroxyl group, being either the nitrogen or oxygen of nitric oxide or the δ-nitrogen of His157.

Table S4. Calculated vs experimental Mössbauer parameters for wild-type **3MPA**-bound MDO with added nitric oxide (percent error shown in parentheses). Calculations are based on bidentate bound **3MPA** with nitrosyl group in the axial position *trans* to His90.

	Isomer shift δ (mm/s)	Quadrupole splitting, $\lvert\Delta E_Q\rvert$ (mm/s)	asymmetry (η)	Ref
Experimental	0.60	1.52	0.4	(6)
Tyr-OH---$N_{(nitrosyl)}$	0.550 (8.4%)	1.474 (3.0%)	0.546	This work
Tyr-OH---$O_{(nitrosyl)}$	0.554 (7.7%)	1.325 (12%)	0.686	This work
Tyr-OH---His157	0.585 (2.5%)	1.611 (6.0%)	0.593	This work

Table S5. The dipolar coupling in MHz and polar angles in degrees for each ^{1}H included in HYSCORE simulations. Shown below are the polar angles that relate a ^{1}H on **3MPA** to magnetic axis system. The angle φ represents rotation in x-y plane, whereas θ is the deviation from the z-axis defined by the Fe-NO bond.

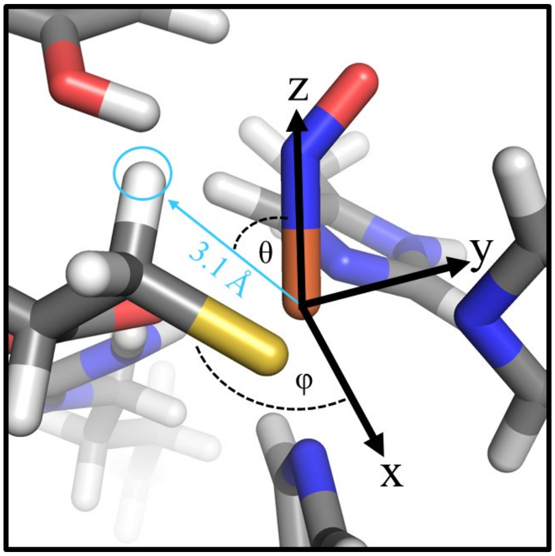

^{1}H	T (MHz)	φ (°)	θ (°)	Distance from Fe (Å)
His90	2.37	35	110	3.4
His90	2.40	160	140	3.4
His92	2.36	110	63	3.3
His92	2.30	80	92	3.5
His142	2.34	40	128	3.5
His142	1.36	165	58	3.3
C3 of **3MPA**	1.16	115	79	4.2
C3 of **3MPA**	2.54	120	59	3.1

SUPPLEMENTAL FIGURES

Figure S1

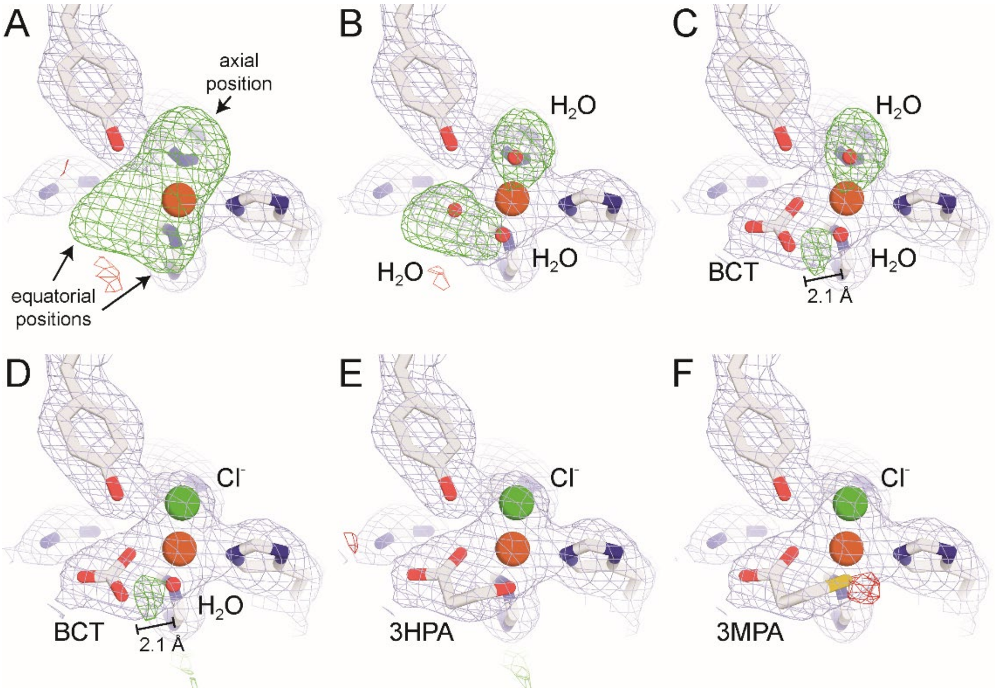

Figure S1. *Av*3MDO active site difference density modeling. In all panels the blue mesh represents sigma A-weighted $2F_o$-F_c density for chain B of the structure contoured at 1 RMSD and the green and red mesh represent NCS-averaged, sigma A-weighted F_o-F_c density contoured at +8 and -8 RMSD respectively. **A**. Map before modeling the open coordination sites. **B**. Updated maps following refinement with three aquo ligands modeled. The three aquo model poorly accounts for the difference map feature. **C**. Updated maps following refinement with two aquo ligands and one bicarbonate (**BCT**) modeled. **D**. Updated maps following refinement with one aquo ligand, one chloride (Cl^-) and bicarbonate modeled. Chloride fully accounts for the difference density at the axial coordination site. Note the unacceptably close contact between bicarbonate and the modeled solvent and the residual strong positive density between these two moieties. **E**. Updated maps following refinement with one chloride (Cl^-) and 3-hydroxypropionic acid (**3HPA**) modeled. This model fully accounts for the active site density. **F**. Updated maps following refinement with one chloride (Cl^-) and 3-mercaptopropionic acid (**3MPA**) modeled. Note the difference map hole at the S atom confirming the presence of a lower *Z* atom (e.g. oxygen) at that site.

Figure S2

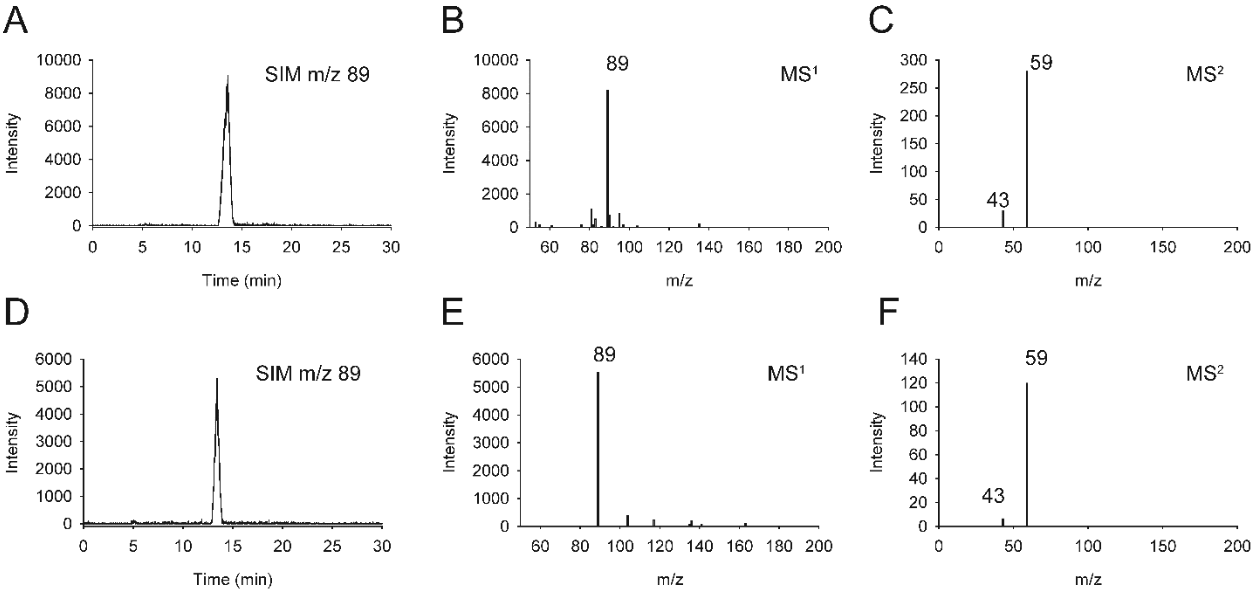

Figure S2. Mass spectrometry detection of **3HPA** in sodium polyacrylate 5100 solution used for *Av*3MDO crystallization. **A.** Negative ion-mode LC-MS of 3HPA standard with selective ion monitoring (SIM) of m/z = 89 species corresponding to deprotonated 3HPA. **B**. MS^1 spectrum of the SIM m/z 89 peak. C) MS^2 spectrum showing fragmentation of the m/z 89 precursor ion to form m/z = 59 (-H_2CO) and 43 (-HCOOH) species. **D**. LC-MS of the polyacrylate solution extract carried out in this same manner as (**A**) showing a single SIM m/z 89 peak with a retention time and fragmentation pattern (panels **E** and **F**) matching those of the 3HPA standard. No SIM m/z 89 peak was observed in control samples with acrylate extract or 3HPA standard omitted. By comparison of the SIM m/z 89 peak areas the concentration of 3HPA in the polyacrylate extract was estimated to be ~20 mM.

Figure S3

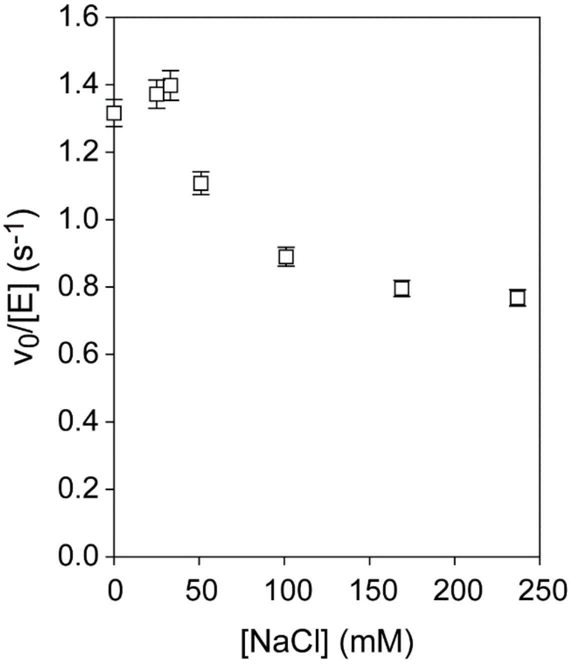

Figure S3. Inhibition of *Av*3MDO catalyzed oxidation of **3MPA** with increasing NaCl concentration. Assays were performed at fixed and saturated **3MPA** concentration (1 mM) in a buffered 20 mM HEPES pH 8 solution at fixed temperature (25 ± 1 °C). Salt concentrations were varied from 0 to 240 mM.

Figure S4

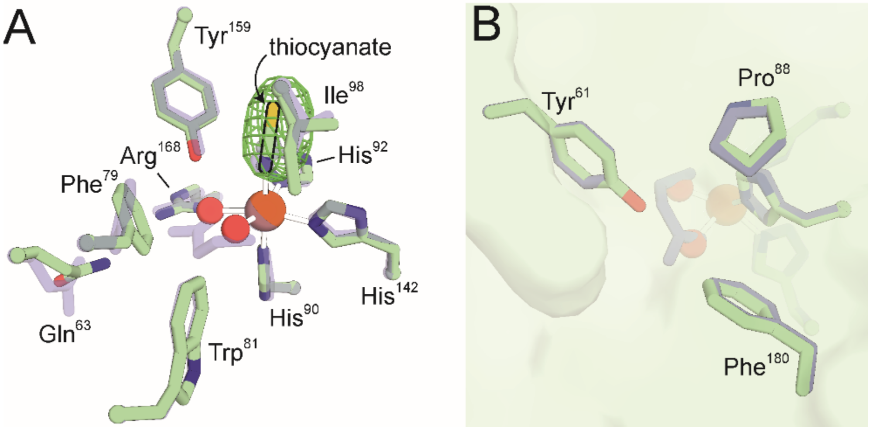

Figure S4. *Crystal structure of Av3MDO in complex with thiocyanate (green) and its comparison to the* ***3HPA****-bound Av3MDO structure (grey).* **A**. Active site comparison between the two structure showing conformational differences in Gln63, Phe79, and Ile98. The green mesh represents unbiased, NCS-averaged, sigma A-weighted Fo-Fc electron density in the axial coordination position supporting the modeling of thiocyanate at this site. **B**. The tunnel providing access to the active site remains occluded in the thiocyanate-bound *Av*3MDO structure with the gatekeeper residues Tyr61, Pro88, and Phe180 found in nearly identical positions as compared to the **3HPA**-bound structure.

Figure S5

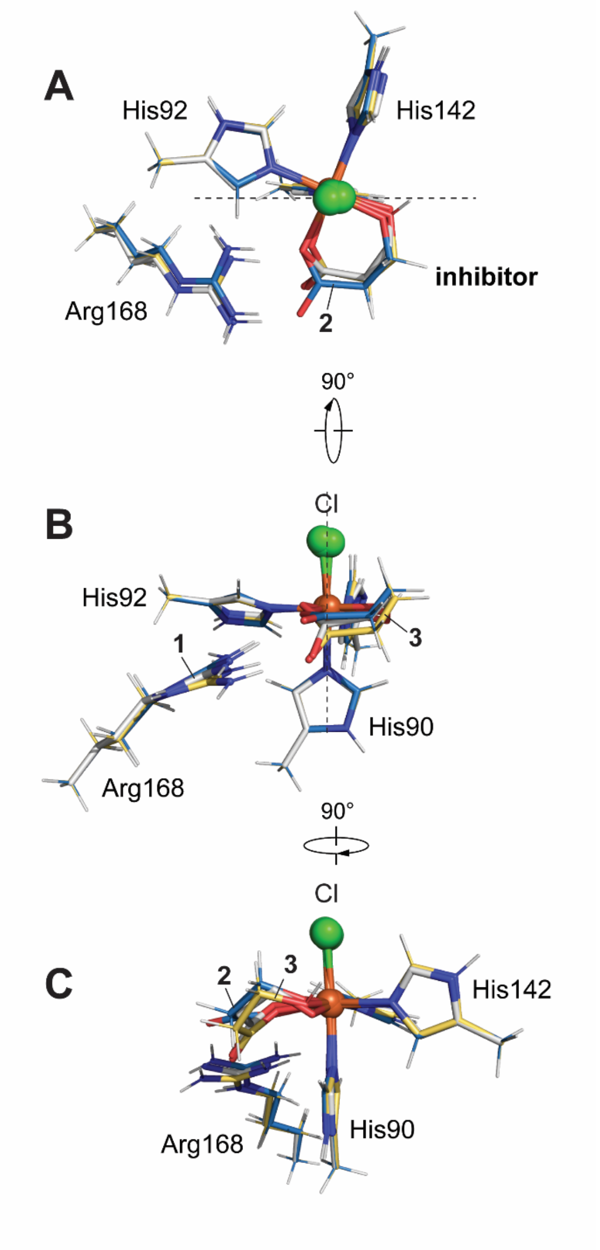

Figure S5. Selected viewpoints (**A**-**C**) of the *Av3MDO*-**3HPA** complex ([**1**], code 6XB9, *shown in white*) overlaid on DFT optimized structures for the **3HPA**-bound Fe-site. Structures for the deprotonated ([**2**], **3HPA**$^{2-}$, *blue carbon atoms*) and protonated ([**3**], **3HPA**$^{1-}$, *yellow carbon atoms*) inhibitor alcohol are presented for comparison. The top-down view shown in panel **A** aligns all structures along the [His90-Fe-Cl] axis. Panel **B** illustrates a 90° rotation of **A** along the perpendicular axis (*dashed line*). Clockwise rotation (-90°) of **B** around the[His90-Fe-Cl] axis results in Panel **C**. Selected distances, angles, and RMSD values are presented in **Table 2** for comparison.

Orientation of Y159 hydrogen bond - Multiple pH-dependent kinetic studies verify that both Tyr159 and His157 of *Av*3MDO are protonated in the catalytically active state (6-8). The crystal structures for the *Av*3MDO-**3HPA** complex presents three possible proton-acceptors for Tyr159. The Nδ-atom of His157 (2.86 Å), the chloride bound to the axial Fe- site (2.85 Å), and the proximal **3HPA**-carboxylate O-atom (3.01 Å). Alternatively, the directionality of the proton relay network could be reversed by Nδ-His157 donation of an H-bond to the Tyr159 phenol O-atom. This leaves Tyr159 free to donate a hydrogen bond to either the axial chloride or the **3HPA**-carboxylate.

Figure S6.

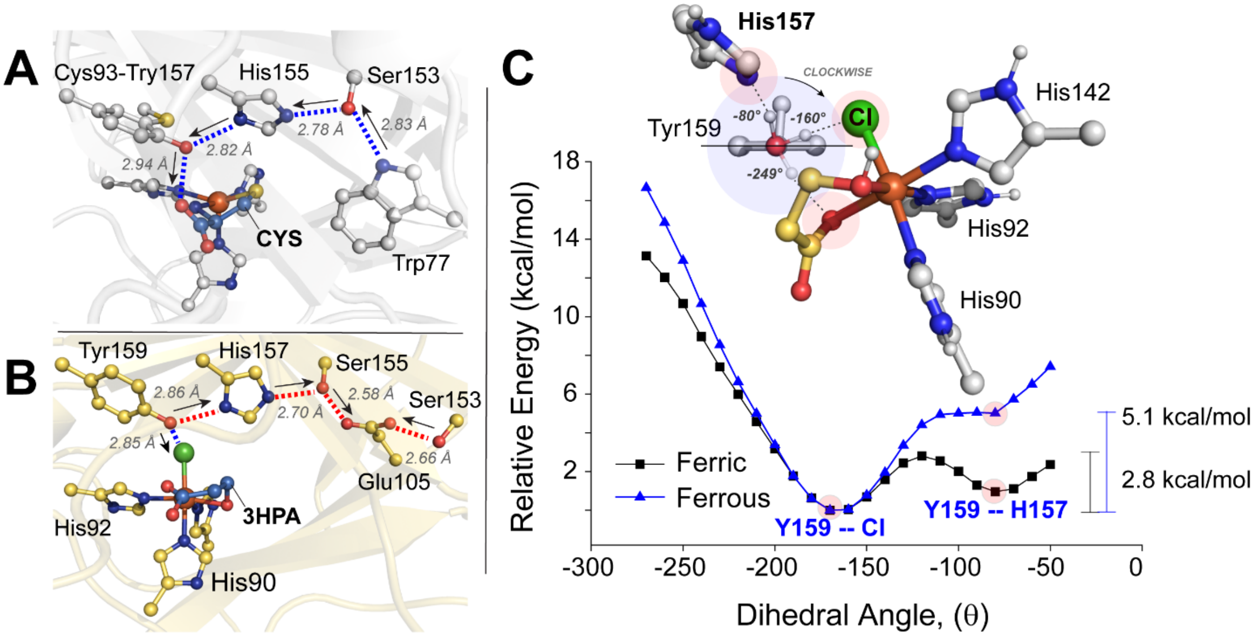

Figure S6. Comparison of **CYS**-bound rat CDO (**A**, PDB 4IEV) and **3HPA**-bound *Av*3MDO (**B**, PDB 6XB9) structures highlighting the extended proton relay network for each enzyme. **C**. Energy surface for Tyr159 H-bond donation/acceptor interactions as a function of dihedral bond angle. Given the ambiguous oxidation state of the Fe-site in the SXB9 structure, relaxed surface scan calculations were performed for both ferrous (*blue*) and ferric (*black*) states.

The energy surface for potential Tyr159 H-bond acceptors was evaluated using a relaxed surface scan (9-11) of the *Av*3MDO-**3HPA** complex. Calculations included Fe-coordinated histidine residues (His90, His92, and His142), the 'SHY' motif (Tyr159, His157, and Ser155), Arg168, **3HPA**$^{-1}$, and chlorine with coordinates taken from the *Av*3MDO crystal structure. All hydrogens were optimized while constraining the crystal structure coordinates. The Tyr159 phenol hydrogen was varied in orientation by varying the dihedral angle defined by the Tyr159 atoms Cε-Cξ-Oη-H. Where Cε denotes the Tyrosine ε-carbon in closest proximity to H157. The path of the relaxed surface scan placed the phenol hydrogen oriented towards the Nδ-atom of His157, axial iron-bound chlorine, and **3HPA**-carboxyl group while keeping the crystal structure rigid. This calculation was done for both Fe(II) and Fe(III) oxidation states to evaluate its influence (if any) on the preferred H-bond orientation. It should be noted that this model does not account for rotation of the tyrosine aromatic ring or other geometric perturbations and thus the values should be considered qualitative.

As illustrated above **(Figure S6C**, *black*), for the ferric state, an activation barrier of 2.8 kcal/mol is calculated for the Tyr159 → His157 configuration from Tyr159 → Cl. Given the small energy barrier and overall energy difference of >1 kcal/mol, it is reasonable that Tyr159 could alternate between donating a hydrogen bond to both His157 and the more preferred iron-bound chlorine. By contrast, the proximal O-atom of the **3HPA**-carboxylate is a poor H-bond acceptor for Tyr159 and is unfavorable in this model. The ferrous state (**Fig**. **S6C**, *blue*) calculations show a more dramatic bias towards Tyr159 → Cl donation. The energy difference between donation towards Cl or His157 is approximately 5 kcal/mol. Qualitatively the activation barrier is also approximately 5 kcal/mol as the Tyr159 → His157 orientation is not in a local minimum. Based on these observations, it can be argued that Tyr159 donates an H-bond to the iron-bound Cl-atom in the *Av*3MDO-**3HPA** complex. Potentially, substitution of the axial chloride ligand for NO could alter the orientation of the Tyr159 H-bond to favor donation to H157. However, this would be inconsistent with previous EPR studies which demonstrate attenuated NO-binding for the Y159F variant (and to a lesser extent H157N). Moreover, as both chlorine and nitrosyl ligands have an equivalent formal charge (-1), an argument can be made that H-bond donation from Tyr159 would similarly favor axially bound NO over His157.

Figure S7

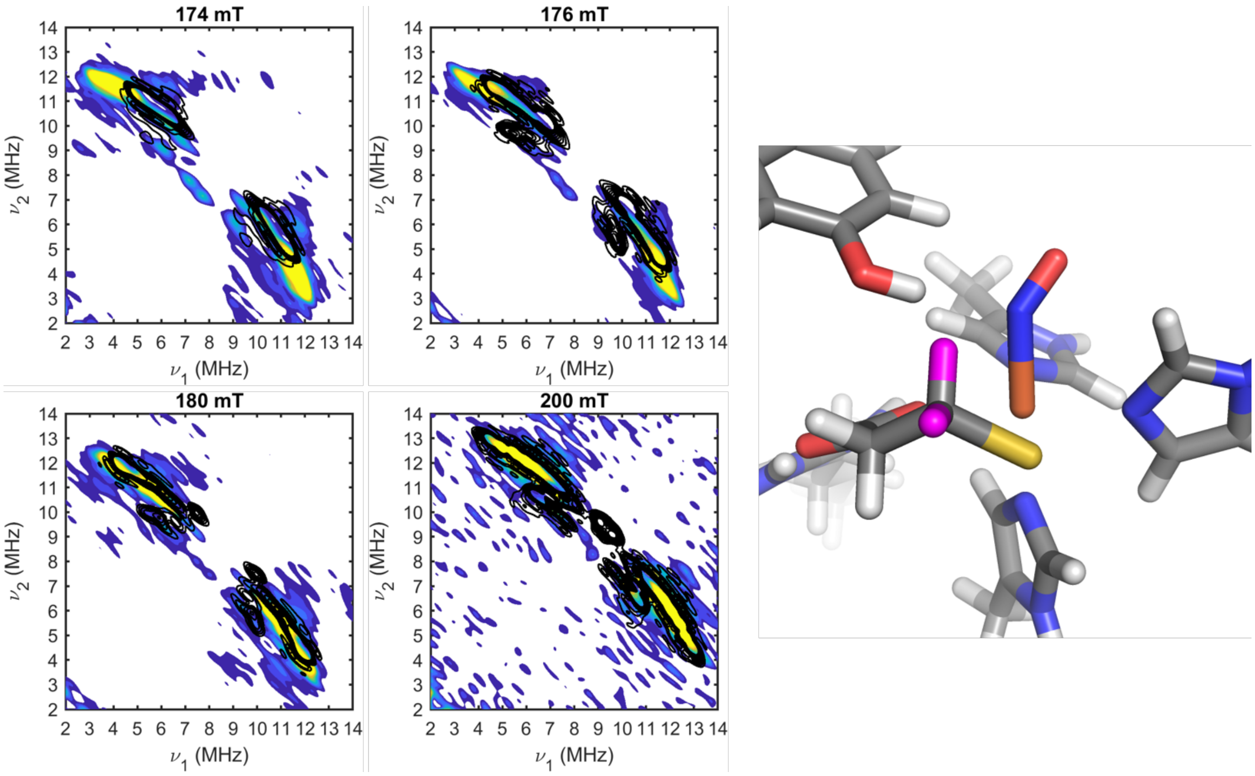

Figure S7. HYSCORE spectra and simulations of the ^{1}H on **3MPA** (*shown in magenta*) at 174, 176, 180, and 200 mT. Experimental spectra appear as color contours; simulations are overlaid as black contour lines. The anisotropic hyperfine couplings and the positions of each nucleus relative to the magnetic axis system are listed in **Table S5**.

Figure S8

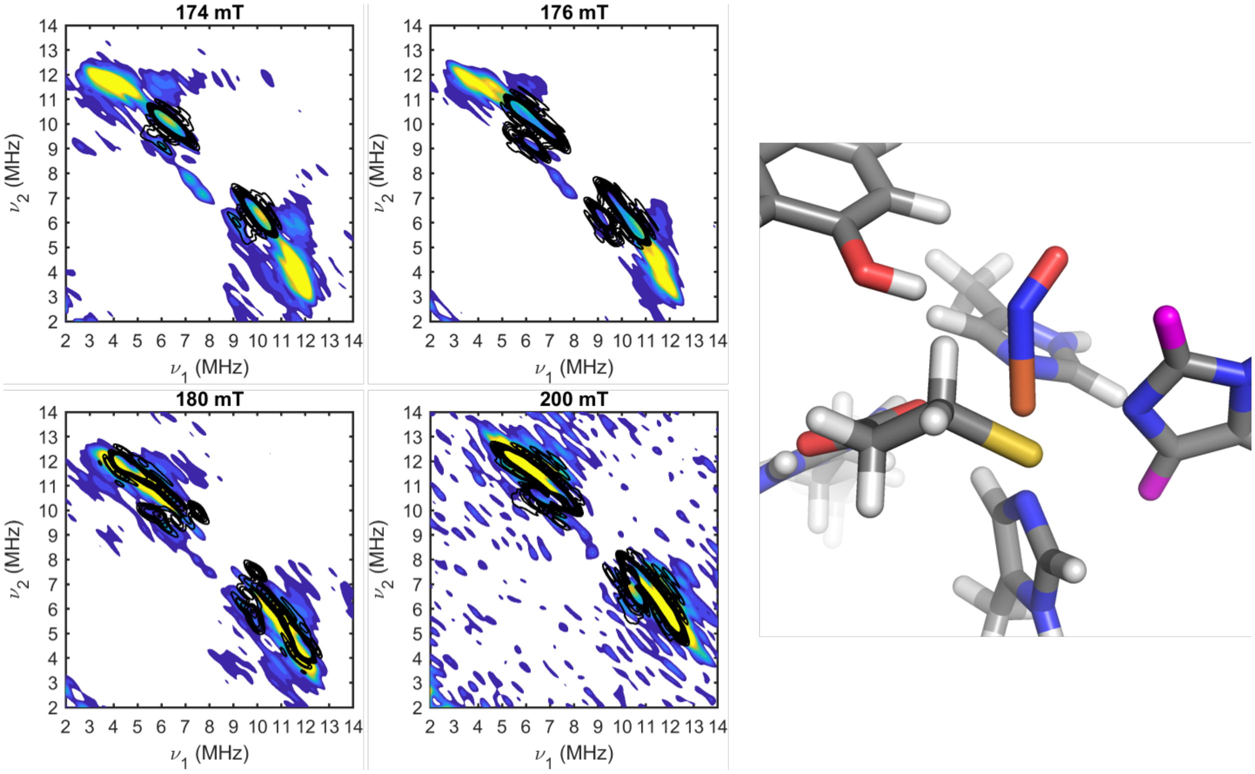

Figure S8. HYSCORE spectra and simulations of the ^{1}H on His142 (*shown in magenta*) at 174, 176, 180, and 200 mT. Experimental spectra appear as color contours; simulations are overlaid as black contour lines. The anisotropic hyperfine couplings and the positions of each nucleus relative to the magnetic axis system are listed in **Table S5**.

Figure S9

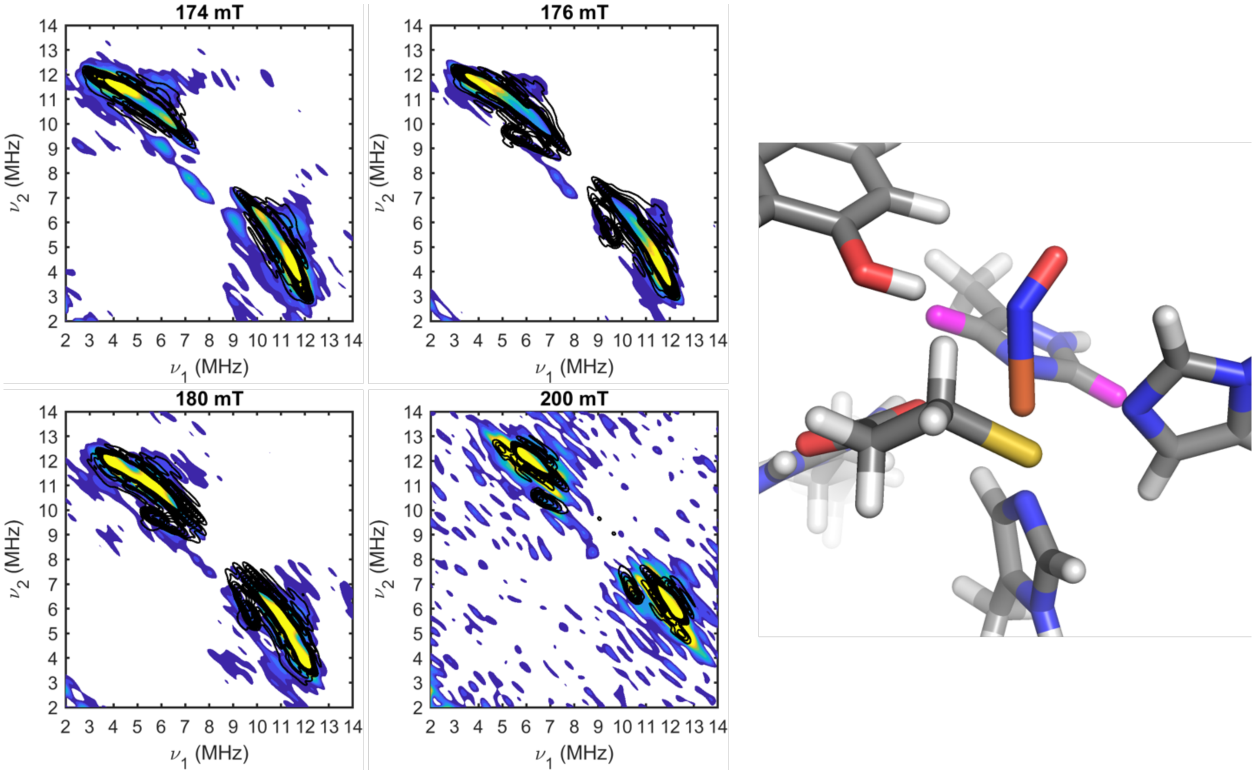

Figure S9. HYSCORE spectra and simulations of the ^{1}H on His92 (*shown in magenta*) at 174, 176, 180, and 200 mT Experimental spectra appear as color contours; simulations are overlaid as black contour lines. The anisotropic hyperfine couplings and the positions of each nucleus relative to the magnetic axis system are listed in **Table S5**.

Figure S10

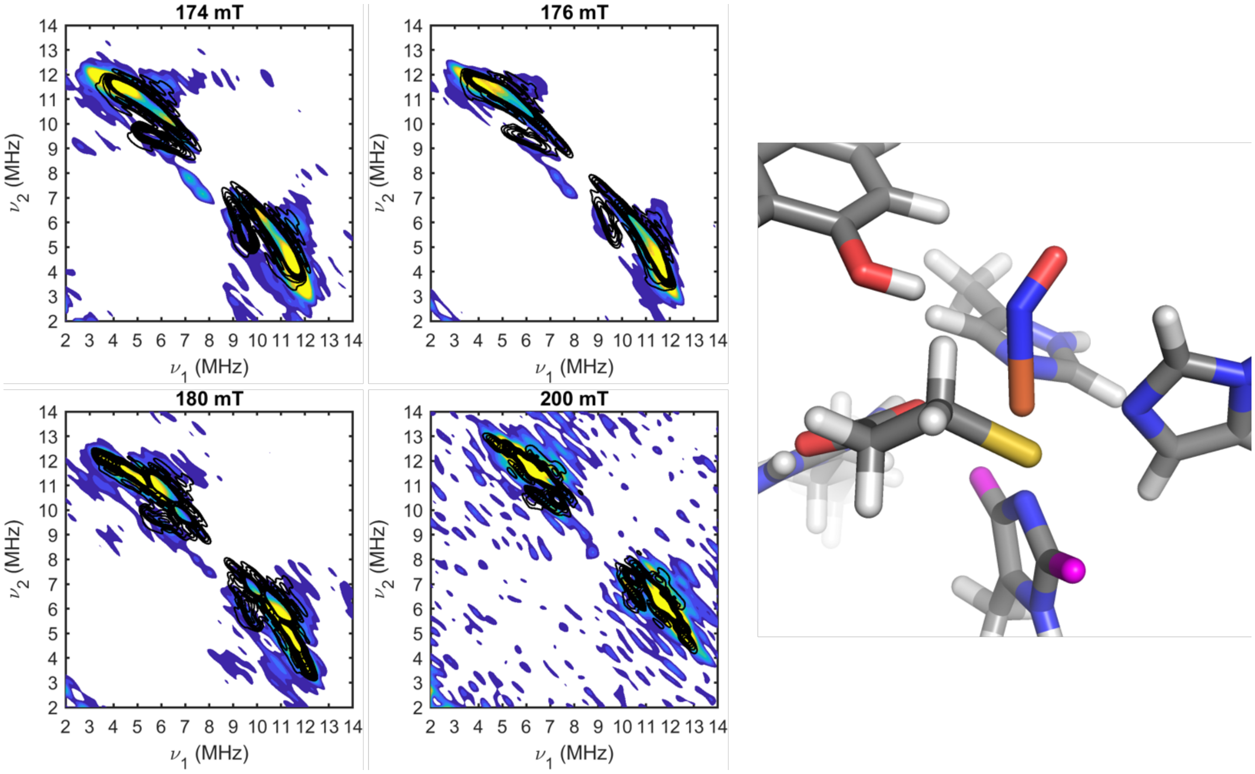

Figure S10. HYSCORE spectra and simulations of the ^{1}H on His90 (*shown in magenta*) at 174, 176, 180, and 200 mT. Experimental spectra appear as color contours; simulations are overlaid as black contour lines. The anisotropic hyperfine couplings and the positions of each nucleus relative to the magnetic axis system are listed in **Table S5**.

Figure S11

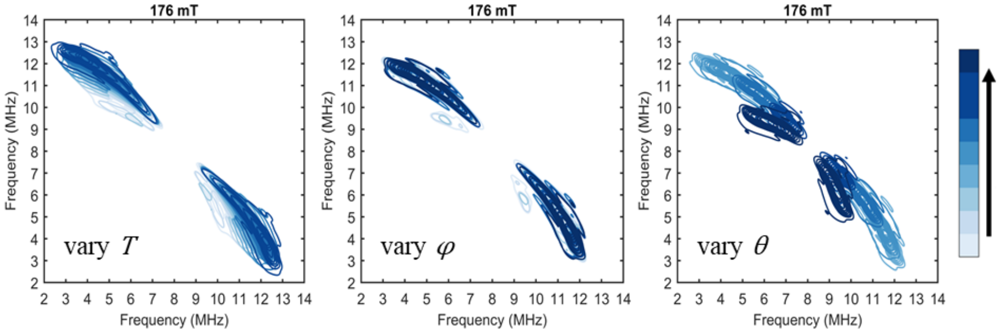

Figure S11. HYSCORE simulations of the closest ^{1}H on **3MPA** at 176 mT. Color gradient represents the simulations that result from increasing the value of T in increments of 0.25 MHz (left) and the angles φ and θ in increments of 15 ° (center and right, respectively).

Figure S12

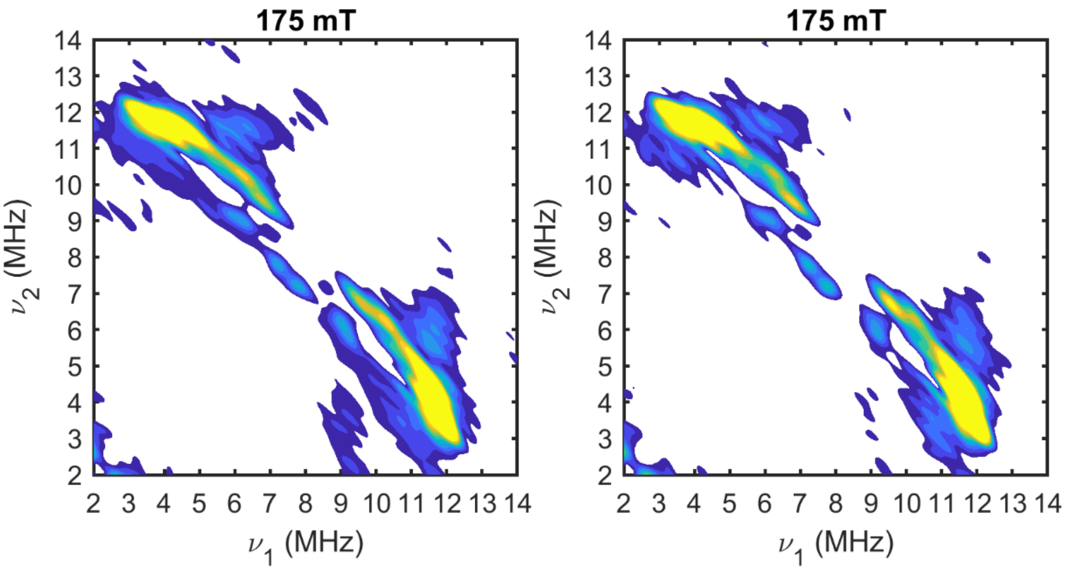

Figure S12. HYSCORE spectra (at 175 mT) for (**3MPA**/NO)-bound *Av*3MDO iron-nitrosyl prepared in ^1H_2O (*left*) buffer as compared to samples exchanged into ^2H_2O buffer (*right*). For clarity, the HYSCORE data is focused entirely on the proton region of the spectra for comparison to ^2H_2O-data. The absence of ^1H peaks lost upon exchange into deuterated buffer suggests no solvent-derived ligands are coordinated to the Fe-site. The expanded HYSCORE spectra of the ^1H_2O data at 175 mT (*left*) is presented in **Fig. 8B**.

Figure S13

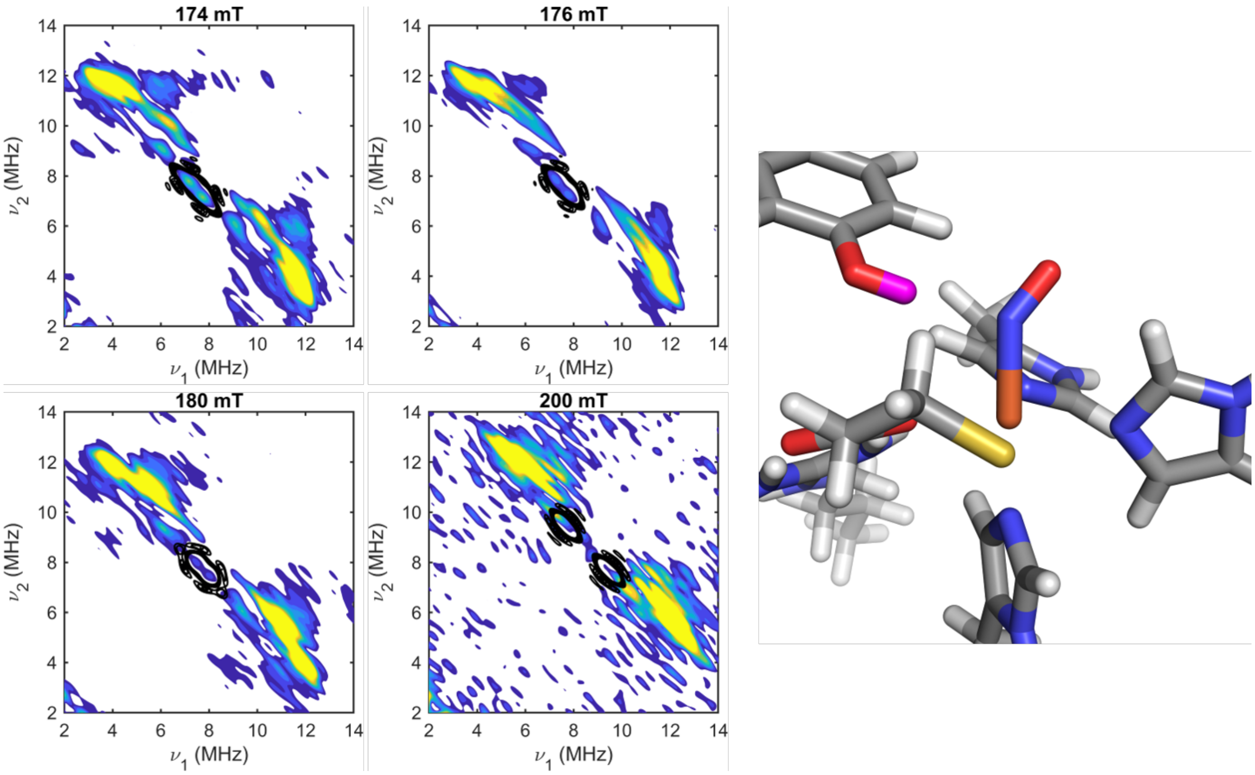

Figure S13. HYSCORE spectra and simulations of the ^{1}H on the hydroxyl group of Tyr159 (*shown in magenta*) at 174, 176, 180, and 200 mT. Experimental spectra appear as color contours; simulations are overlaid as black contour lines. The simulations were calculated with an anisotropic hyperfine coupling of 0.95 MHz and values of 40° and 20° for θ and φ, respectively.

CARTESIAN COORDINATES (Å) FOR SELECTED MODELS

3MPA-bound Fe(III) MDO: **Figure 6A-B (*white*)**

H	-0.650665	5.196980	-3.480381
C	0.043044	4.393511	-3.810041
C	0.389368	3.454927	-2.704539
C	-0.294929	2.374957	-2.170192
N	1.560951	3.531272	-1.964934
C	1.567036	2.530631	-1.042361
N	0.446082	1.816556	-1.144985
H	0.947061	4.879654	-4.227719
H	-0.458354	3.851628	-4.635215
H	-1.260255	1.966679	-2.471889
H	2.309613	4.215469	-2.100812
H	2.380602	2.331782	-0.337970
H	-5.145542	-0.170879	-3.619917
C	-5.125358	-0.719738	-2.653956
C	-4.017623	-0.204012	-1.801587
C	-2.731610	-0.659407	-1.545621
N	-4.108009	1.002045	-1.119387
C	-2.925449	1.241877	-0.489648
N	-2.071309	0.254229	-0.740353
H	-5.008614	-1.794365	-2.893642
H	-6.117770	-0.588111	-2.177323
H	-2.250104	-1.588981	-1.861295
H	-4.935763	1.601724	-1.077851
H	-2.726646	2.123190	0.127659
H	-1.549177	5.264368	2.640892
C	-1.720819	4.456978	3.377330
C	-1.336438	3.123773	2.824346
C	-0.760310	2.731605	1.624042
N	-1.544688	1.937566	3.517589
C	-1.116329	0.895507	2.756643
N	-0.637168	1.349298	1.600343
H	-2.796119	4.469168	3.655434
H	-1.141414	4.704893	4.290318
H	-0.430794	3.351783	0.786380
H	-1.950325	1.856838	4.453096
H	-1.169297	-0.158097	3.047803
H	-4.631338	1.817273	-10.348492
C	-4.447775	2.512029	-9.502023
H	-3.613703	3.183515	-9.797597
C	-4.178612	1.816575	-8.156824
C	-2.860806	1.036462	-8.050456

C	-2.636147	0.473823	-6.638548
N	-1.398117	-0.299709	-6.578682
C	-0.651136	-0.547695	-5.479535
N	-1.075400	-0.165815	-4.253644
N	0.533585	-1.151735	-5.593797
H	-5.354836	3.138412	-9.381738
H	-5.023670	1.131860	-7.920623
H	-4.189076	2.591057	-7.357155
H	-2.003788	1.694692	-8.313339
H	-2.855304	0.196659	-8.781416
H	-3.507389	-0.155014	-6.340109
H	-2.565902	1.308024	-5.907474
H	-1.085540	-0.740192	-7.446489
H	-0.494670	-0.400469	-3.425668
H	-2.071670	-0.035289	-4.075433
H	0.951289	-1.282651	-6.515171
H	1.087911	-1.377710	-4.716377
Fe	0.000000	0.000000	0.000000
C	1.724416	-1.369261	-2.177822
O	0.581565	-0.924913	-1.773860
C	2.891318	-1.582200	-1.216953
O	1.952429	-1.644411	-3.383123
C	2.619212	-1.656163	0.282256
S	2.075014	-0.077732	1.023478
H	3.383131	-2.521430	-1.546960
H	3.551109	-1.954210	0.802075
H	1.844346	-2.416364	0.510057
H	3.622706	-0.773104	-1.433495
Cl	-0.815983	-1.998656	0.951838

3MPA-bound Fe(II) MDO: **Figure 6C-D (*white*)**

H	-0.659818	5.172145	-3.543928
C	0.033890	4.368677	-3.873587
C	0.400611	3.410832	-2.787567
C	-0.360249	2.517794	-2.049616
N	1.706147	3.185966	-2.372522
C	1.704899	2.197730	-1.433411
N	0.461088	1.777664	-1.219723
H	0.937908	4.854820	-4.291266
H	-0.467507	3.826793	-4.698762
H	-1.439078	2.349165	-2.082939
H	2.536057	3.664863	-2.728537
H	2.588319	1.783644	-0.934540

H	-5.154695	-0.195713	-3.683464
C	-5.134511	-0.744572	-2.717503
C	-4.021256	-0.251300	-1.856721
C	-2.697586	-0.647659	-1.708352
N	-4.151498	0.858212	-1.033404
C	-2.950565	1.093123	-0.427569
N	-2.052460	0.199686	-0.827221
H	-5.017768	-1.819199	-2.957189
H	-6.126923	-0.612945	-2.240870
H	-2.176432	-1.500512	-2.152987
H	-5.010846	1.391415	-0.884704
H	-2.772779	1.904596	0.285208
H	-1.558330	5.239533	2.577346
C	-1.729973	4.432144	3.313783
C	-1.346636	3.090646	2.781716
C	-0.739490	2.674166	1.604885
N	-1.583726	1.920150	3.489786
C	-1.135967	0.861032	2.750189
N	-0.622915	1.291369	1.601905
H	-2.805272	4.444333	3.591887
H	-1.150567	4.680059	4.226771
H	-0.385250	3.274663	0.761954
H	-2.009084	1.859371	4.416928
H	-1.212206	-0.190542	3.045725
H	-4.640491	1.792439	-10.412039
C	-4.456928	2.487194	-9.565570
H	-3.622856	3.158681	-9.861144
C	-4.168938	1.797033	-8.220691
C	-2.781148	1.156292	-8.096671
C	-2.500119	0.599871	-6.692634
N	-1.152895	0.045491	-6.638860
C	-0.398915	-0.186103	-5.533327
N	-0.852690	0.072815	-4.296496
N	0.838305	-0.683208	-5.674836
H	-5.363989	3.113578	-9.445285
H	-4.950211	1.032129	-8.014562
H	-4.271630	2.555542	-7.412401
H	-1.998026	1.908634	-8.337513
H	-2.670484	0.335519	-8.840867
H	-3.263773	-0.167810	-6.425424
H	-2.583756	1.419703	-5.945313
H	-0.751642	-0.268072	-7.524425
H	-0.265227	-0.231392	-3.463350

H	-1.837958	0.276042	-4.133880
H	1.246318	-0.701712	-6.610394
H	1.497545	-0.622518	-4.818350
Fe	0.000000	0.000000	0.000000
C	1.930677	-0.981455	-2.503181
O	0.689135	-0.970618	-2.213910
C	2.946857	-1.653098	-1.579882
O	2.413439	-0.501704	-3.582700
C	2.533572	-1.899798	-0.134337
S	2.171590	-0.346624	0.773161
H	3.194433	-2.621831	-2.076482
H	3.348165	-2.445754	0.386958
H	1.623401	-2.532142	-0.088783
H	3.876377	-1.049110	-1.623223
Cl	-0.932213	-2.076951	0.959885

3HPA^{-1}-bound Fe(III) MDO: **Figure S4 [3]**

H	-0.512996	5.123085	-3.483805
C	0.181136	4.323343	-3.819805
C	0.512946	3.391293	-2.701982
C	-0.174119	2.312246	-2.165755
N	1.655287	3.503223	-1.922860
C	1.647362	2.532805	-0.971914
N	0.539365	1.797654	-1.095684
H	1.085671	4.811009	-4.233053
H	-0.320321	3.781379	-4.644189
H	-1.130164	1.886705	-2.476802
H	2.395672	4.199273	-2.049713
H	2.433323	2.385609	-0.226363
H	-4.997627	-0.243256	-3.624668
C	-4.981542	-0.795032	-2.660837
C	-3.882057	-0.297029	-1.786774
C	-2.562151	-0.689687	-1.615613
N	-4.040698	0.796256	-0.945156
C	-2.876595	1.040051	-0.298290
N	-1.958755	0.152612	-0.692119
H	-4.864180	-1.869148	-2.900769
H	-5.972215	-0.659890	-2.182810
H	-2.017194	-1.523128	-2.067573
H	-4.907517	1.326272	-0.818218
H	-2.725220	1.845678	0.426076
H	-1.410421	5.194799	2.633177
C	-1.580171	4.386388	3.368195

C	-1.166585	3.057143	2.836376
C	-0.605267	2.655782	1.635427
N	-1.296715	1.886961	3.575512
C	-0.843633	0.841613	2.845793
N	-0.413200	1.276802	1.654695
H	-2.655304	4.390544	3.645032
H	-1.001421	4.629013	4.282392
H	-0.336554	3.263503	0.769013
H	-1.674703	1.820361	4.524847
H	-0.847708	-0.200792	3.176875
H	-4.493511	1.740848	-10.355650
C	-4.309805	2.436095	-9.509782
H	-3.476660	3.108239	-9.806178
C	-4.008639	1.696561	-8.202399
C	-2.718933	0.868204	-8.233751
C	-2.456576	0.169469	-6.896131
N	-1.196847	-0.571516	-6.916130
C	-0.309104	-0.630861	-5.907795
N	-0.603713	-0.150976	-4.684759
N	0.908846	-1.163694	-6.113153
H	-5.217429	3.061288	-9.388605
H	-4.863044	1.031218	-7.945322
H	-3.938419	2.434686	-7.372041
H	-1.852333	1.522851	-8.472788
H	-2.778230	0.106298	-9.043022
H	-3.300505	-0.512048	-6.646026
H	-2.394200	0.929439	-6.089695
H	-0.951815	-1.056695	-7.782849
H	0.155138	-0.219779	-3.973730
H	-1.573684	-0.063029	-4.384145
H	1.265012	-1.312792	-7.057130
H	1.556798	-1.223173	-5.319517
Fe	0.000000	0.000000	0.000000
C	1.824473	-0.732165	-2.200324
O	0.705661	-0.949192	-1.573223
C	3.108370	-0.738706	-1.382936
O	1.870952	-0.524933	-3.427259
C	2.940822	-1.010481	0.099010
O	2.125411	0.056650	0.680625
H	3.773436	-1.513202	-1.818321
H	3.925780	-1.008222	0.605332
H	2.442167	-1.984384	0.279905
H	3.620202	0.230907	-1.554935
Cl	-0.334927	-1.907827	1.206628
H	2.098497	-0.077710	1.649766

(**3MPA**/NO)-bound *Av*3MDO: **Figure 7**

H	-0.657168	5.182547	-3.470783
C	0.037195	4.377758	-3.796259
C	0.367124	3.468050	-2.666676
C	-0.205132	2.288917	-2.251554
N	1.363176	3.723848	-1.739865
C	1.377406	2.718439	-0.831737
N	0.433065	1.839845	-1.114371
H	0.942132	4.862872	-4.213494
H	-0.462560	3.834405	-4.621596
H	-1.015569	1.736086	-2.709643
H	1.992852	4.520562	-1.743532
H	2.078191	2.650176	-0.004467
H	-5.153299	-0.186197	-3.610683
C	-5.134348	-0.733048	-2.643426
C	-4.032284	-0.221588	-1.787813
C	-2.747398	-0.660680	-1.548337
N	-4.137655	0.964107	-1.087555
C	-2.960471	1.208300	-0.466477
N	-2.096413	0.248524	-0.735998
H	-5.016000	-1.807506	-2.882765
H	-6.128073	-0.602434	-2.169123
H	-2.258101	-1.566562	-1.893568
H	-4.965913	1.548737	-1.031276
H	-2.781305	2.074240	0.161597
H	-1.568815	5.259027	2.650252
C	-1.741950	4.452357	3.387237
C	-1.321639	3.125479	2.851428
C	-0.901586	2.713012	1.606545
N	-1.294415	1.979792	3.625780
C	-0.882994	0.943324	2.861753
N	-0.639433	1.352270	1.630606
H	-2.817877	4.465849	3.663360
H	-1.164213	4.702319	4.300884
H	-0.770141	3.307447	0.709429
H	-1.542540	1.919426	4.608444
H	-0.772007	-0.070348	3.234403
H	-3.955304	-8.732796	-1.303464
C	-4.645789	-8.042482	-1.832776
H	-5.438392	-8.657832	-2.307857

C	-3.928354	-7.187393	-2.886579
C	-2.824045	-6.306712	-2.342035
C	-3.099846	-5.199012	-1.522854
C	-1.476862	-6.575671	-2.632917
C	-2.077016	-4.396779	-1.008233
C	-0.442891	-5.781498	-2.134361
C	-0.736444	-4.683865	-1.312840
O	0.299717	-3.946628	-0.835528
H	-5.130242	-7.407497	-1.061355
H	-4.679693	-6.556765	-3.395345
H	-3.504434	-7.846557	-3.662129
H	-4.136123	-4.950626	-1.271770
H	-1.229080	-7.428499	-3.271241
H	-2.320294	-3.548325	-0.364797
H	0.599797	-5.998786	-2.377965
H	-0.027109	-3.149316	-0.383425
H	-4.623996	1.792135	-10.340640
C	-4.441755	2.488241	-9.494928
H	-3.606491	3.158312	-9.790493
C	-4.187142	1.787307	-8.155582
C	-2.850467	1.054512	-8.047236
C	-2.632741	0.439244	-6.662923
N	-1.349466	-0.251570	-6.622493
C	-0.639863	-0.584185	-5.532909
N	-1.121874	-0.360550	-4.299454
N	0.565238	-1.125586	-5.662115
H	-5.348558	3.115593	-9.377538
H	-5.007542	1.076813	-7.946949
H	-4.238964	2.545320	-7.352799
H	-2.021900	1.752472	-8.260476
H	-2.792103	0.253656	-8.807243
H	-3.457047	-0.258283	-6.423829
H	-2.642459	1.235060	-5.898156
H	-0.964522	-0.547953	-7.513132
H	-0.569424	-0.626992	-3.474119
H	-2.101282	-0.153673	-4.156210
H	0.990201	-1.219386	-6.576318
H	1.069980	-1.434441	-4.802797
Fe	0.000000	0.000000	0.000000
C	1.576968	-1.558369	-2.169151
O	0.496813	-0.999314	-1.751462
C	2.758115	-1.829645	-1.240526
O	1.734304	-1.886774	-3.362887

C	2.579796	-1.768826	0.271909
S	2.187014	-0.108480	0.928069
H	3.132915	-2.825747	-1.526138
H	3.520971	-2.093323	0.740307
H	1.809685	-2.480413	0.602968
H	3.540881	-1.113480	-1.544318
N	-0.677029	-1.559548	0.836412
O	-1.488359	-1.853049	1.646726

REFERENCES.

1. Driggers, C. M., Stipanuk, M. H., and Karplus, P. A. (2015) Mammalian cysteine dioxygenase. in *Encyclopedia of Inorganic and Bioinorganic Chemistry* (Scott, R. A. ed., John Wiley & Sons, Ltd.
2. Fischer, A. A., Miller, J. R., Jodts, R. J., Ekanayake, D. M., Lindeman, S. V., Brunold, T. C., and Fiedler, A. T. (2019) Spectroscopic and Computational Comparisons of Thiolate-Ligated Ferric Nonheme Complexes to Cysteine Dioxygenase: Second-Sphere Effects on Substrate (Analogue) Positioning. *Inorg Chem.* **58**, 16487-16499
3. Fischer, A. A., Stracey, N., Lindeman, S. V., Brunold, T. C., and Fiedler, A. T. (2016) Synthesis, X-ray Structures, Electronic Properties, and O2/NO Reactivities of Thiol Dioxygenase Active-Site Models. *Inorg Chem.* **55**, 11839-11853
4. Gordon, J. B., McGale, J. P., Prendergast, J. R., Shirani-Sarmazeh, Z., Siegler, M. A., Jameson, G. N. L., and Goldberg, D. P. (2018) Structures, Spectroscopic Properties, and Dioxygen Reactivity of 5- and 6-Coordinate Nonheme Iron(II) Complexes: A Combined Enzyme/Model Study of Thiol Dioxygenases. *J Am Chem Soc.* **140**, 14807-14822
5. Li, W., Blaesi, E. J., Pecore, M. D., Crowell, J. K., and Pierce, B. S. (2013) Second-Sphere Interactions between the C93–Y157 Cross-Link and the Substrate-Bound Fe Site Influence the O2 Coupling Efficiency in Mouse Cysteine Dioxygenase. *Biochemistry* **52**, 9104-9119
6. Sardar, S., Weitz, A., Hendrich, M. P., and Pierce, B. S. (2019) Outer-Sphere Tyrosine 159 within the 3-Mercaptopropionic Acid Dioxygenase S-H-Y Motif Gates Substrate-Coordination Denticity at the Non-Heme Iron Active Site. *Biochemistry* **58**, 5135-5150
7. Crowell, J. K., Sardar, S., Hossain, M. S., Foss Jr, F. W., and Pierce, B. S. (2016) Non-chemical proton-dependent steps prior to O2-activation limit Azotobacter vinelandii 3-mercaptopropionic acid dioxygenase (MDO) catalysis. *Arch Biochem Biophys.* **604**, 86-94
8. Pierce, B. S., Subedi, B. P., Sardar, S., and Crowell, J. K. (2015) The “Gln-Type” Thiol Dioxygenase from Azotobacter vinelandii Is a 3-Mercaptopropionic Acid Dioxygenase. *Biochemistry* **54**, 7477-7490
9. Dajnowicz, S., Parks, J. M., Hu, X., Gesler, K., Kovalevsky, A. Y., and Mueser, T. C. (2017) Direct evidence that an extended hydrogen-bonding network influences activation of pyridoxal 5′-phosphate in aspartate aminotransferase. *J Biol Chem.* **292**, 5970-5980
10. Bykov, D., Plog, M., and Neese, F. (2014) Heme-bound nitroxyl, hydroxylamine, and ammonia ligands as intermediates in the reaction cycle of cytochrome c nitrite reductase: a theoretical study. *J Biol Inorg Chem* **19**, 97-112
11. Kampa, M., Lubitz, W., van Gastel, M., and Neese, F. (2012) Computational study of the electronic structure and magnetic properties of the Ni–C state in [NiFe] hydrogenases including the second coordination sphere. *J Biol Inorg Chem* **17**, 1269-1281
